# Transcriptomic Evidence for a Dramatic Functional Transition of the Malpighian Tubules after a Blood Meal in the Asian Tiger Mosquito *Aedes albopictus*

**DOI:** 10.1371/journal.pntd.0002929

**Published:** 2014-06-05

**Authors:** Carlos J. Esquivel, Bryan J. Cassone, Peter M. Piermarini

**Affiliations:** 1 Department of Entomology, Ohio Agricultural Research and Development Center, The Ohio State University, Wooster, Ohio, United States of America; 2 Department of Plant Pathology, Ohio Agricultural Research and Development Center, The Ohio State University, Wooster, Ohio, United States of America; National Institute of Allergy and Infectious Diseases, United States of America

## Abstract

**Background:**

The consumption of a vertebrate blood meal by adult female mosquitoes is necessary for their reproduction, but it also presents significant physiological challenges to mosquito osmoregulation and metabolism. The renal (Malpighian) tubules of mosquitoes play critical roles in the initial processing of the blood meal by excreting excess water and salts that are ingested. However, it is unclear how the tubules contribute to the metabolism and excretion of wastes (e.g., heme, ammonia) produced during the digestion of blood.

**Methodology/Principal Findings:**

Here we used RNA-Seq to examine global changes in transcript expression in the Malpighian tubules of the highly-invasive Asian tiger mosquito *Aedes albopictus* during the first 24 h after consuming a blood meal. We found progressive, global changes in the transcriptome of the Malpighian tubules isolated from mosquitoes at 3 h, 12 h, and 24 h after a blood meal. Notably, a DAVID functional cluster analysis of the differentially-expressed transcripts revealed 1) a down-regulation of transcripts associated with oxidative metabolism, active transport, and mRNA translation, and 2) an up-regulation of transcripts associated with antioxidants and detoxification, proteolytic activity, amino-acid metabolism, and cytoskeletal dynamics.

**Conclusions/Significance:**

The results suggest that blood feeding elicits a functional transition of the epithelium from one specializing in active transepithelial fluid secretion (e.g., diuresis) to one specializing in detoxification and metabolic waste excretion. Our findings provide the first insights into the putative roles of mosquito Malpighian tubules in the chronic processing of blood meals.

## Author Summary

The Asian tiger mosquito *Aedes albopictus* is a vector of several medically-important arboviruses and one of the most invasive mosquito species in the world. Existing control measures for mosquitoes are presently being challenged by the emergence of resistance to insecticides that target the nervous system. Thus, it is necessary to identify novel physiological targets to guide the development of new insecticides. We recently demonstrated that the ‘kidneys’ (Malpighian tubules) of mosquitoes offer a valuable, new physiological target for insecticides. However, our understanding of how this tissue contributes to the chronic metabolic processing of blood meals by mosquitoes is limited. Here we characterize the changes in transcript expression that occur in the Malpighian tubules of adult female *A. albopictus* with the goal of identifying key molecular pathways that may reveal valuable targets for insecticide development. We find dramatic changes in transcript accumulation in Malpighian tubules, which 1) provide new insights into the potential functional roles of Malpighian tubules after a blood meal, and 2) reveal new potential molecular pathways and targets to guide the development of new insecticides that would disrupt the renal functions of mosquitoes.

## Introduction

The Asian tiger mosquito *Aedes albopictus* is considered one of the most invasive mosquito species in the world; since 1979 it has spread to over 28 countries outside of its native range in Asia and Southeast Asia, aided by the international trade of used automobile tires [1], [2]. Within the United States, the mosquito has spread to at least 36 states and models of its potential for range expansion in the northeastern United States within the next few decades are alarming [3]. Moreover, this species is a known or suspected vector of several medically important arboviruses, including chikungunya, dengue, eastern equine encephalitis, La Crosse, West Nile, and yellow fever [4]. Thus, *A. albopictus* is an emerging threat to global health for which effective control measures need to be developed.

Historically, mosquitoes have been controlled through the use of insecticides that target the nervous system (e.g., carbamates, organophosphates, organochlorines, and pyrethroids). However, resistance to these control agents is limiting their efficacy. In particular, the yellow fever mosquito *Aedes aegypti* exhibits high levels of resistance to insecticides in certain parts of the world, and there is concern that *A. albopictus* will soon develop such resistance [5]. Thus, it is important to identify new control agents that target novel physiological systems in mosquitoes to help combat the emerging threat of insecticide resistance.

A recent study by our group demonstrated that the renal excretory system (Malpighian tubules) of mosquitoes represents a valuable new physiological target for insecticides [6]. The Malpighian tubules produce urine via transepithelial fluid secretion, which is mediated by the coordinated actions of a V-type H^+^-ATPase along with several ion transporters, ion channels, and water channels [7]. In adult female *A. aegypti* mosquitoes, the Malpighian tubules play an especially important role in the post-prandial diuresis when the mosquito excretes urine during and after the engorgement of vertebrate blood [8]. The diuresis lasts for up to two hours after feeding and excretes a significant fraction of the ingested Na^+^, K^+^, Cl^−^, and water from the blood [8]. Once this diuresis ends, the female mosquito will continue to digest and metabolize the blood meal over the next two days to nourish the development of her eggs. The physiological importance of the Malpighian tubules during this time is unknown, but they presumably play a critical role in excreting excess nitrogenous wastes and other metabolites that are generated during the processing of the protein-rich meal [9], especially within the first 24 hours when ∼75–90% of the ingested protein is digested [10], [11].

Other groups have documented the effects of ingesting blood on the transcriptomes of adult female *A. aegypti* and *Anopheles gambiae* mosquitoes [12], [13], [14], [15], [16], including more focused studies on how blood feeding influences tissue-specific transcriptomes in the antennae, fat body, midgut, and salivary glands of these species [17], [18], [19], [20]. However, no previous studies have examined the effects of blood-feeding on the transcriptome of mosquito Malpighian tubules.

The goal of the present study was to characterize the global changes in transcript expression that occur in the Malpighian tubules of *A. albopictus* during the first 24 h after female mosquitoes consume a blood meal (using RNA-Seq), with the aim of identifying key metabolic pathways and transcripts that are activated or suppressed in the renal tubules during the processing of the blood meal. We found that blood feeding elicits dramatic, time-dependent changes to the Malpighian-tubule transcriptome of *A. albopictus*. A functional cluster analysis of the differentially-expressed transcripts revealed a potential functional transition of the tubule epithelium after blood feeding from one specializing in active transepithelial fluid secretion to one specializing in detoxification and metabolic waste excretion.

## Methods

### Mosquito colony

*A. albopictus* eggs were obtained from the Malaria Research and Reference Reagent Resource Center (MR4) as part of BEI Resources Repository, NIAID, NIH (ALBOPICTUS, MRA-804, deposited by Sandra Allan). Eggs were raised to adults using a protocol similar to that described for *A. aegypti* [21] with the exception that larvae were fed pulverized TetraMin flakes (Melle, Germany). Adult females between 5 to 10 days post-eclosion were used for the present study.

### Design of the study

The experimental design consisted of two treatments, blood fed (BF) and non-blood fed (NBF) females at three different time points. In brief, the BF mosquitoes were fed on heparinized rabbit blood for 30 minutes (see details below) and collected at 3 h, 12 h, or 24 h after feeding. These time points occur after the post-prandial diuresis, which ends within 2 h after feeding [8]. Moreover, one or more of these time points has been commonly used in other studies examining the effects of blood feeding on gene expression in mosquitoes [13], [14], [15], [16], [17], [18], [19], [20], [22]. NBF females were only offered a 10% sucrose solution and dissected at similar time points to serve as controls. Each treatment/time point was replicated three times using females from different cohorts (i.e., 3 biological replicates per time point).

### Blood feeding and isolation of Malpighian tubules

For each experimental treatment, 90 adult female mosquitoes were transferred to two small 32 oz. cages (45 females per cage) without access to a sucrose solution for 24 h prior to offering them blood or sucrose. To one cage, a membrane feeder (Hemotek, Blackburn, UK) was used to feed the mosquitoes warmed blood (37°C), which consisted of heparinized rabbit blood (purchased from Hemostat Laboratories, Dixon, CA) supplemented with adenosine 5′-triphosphoric acid (disodium salt; Sigma, St. Louis, MO) at a concentration of 0.01 g/ml. A solution of 10% lactic acid was applied to the membrane surface as an attractant. Females were given access to the blood for a period of 30 min before the feeder was removed from the cage. In the other cage, the mosquitoes were given access to cotton balls soaked with 10% sucrose for 30 min. At 3 h, 12 h, or 24 h after removing the feeder or cotton balls, the cage was refrigerated on ice to immobilize the mosquitoes.

Before dissecting the mosquitoes that were offered blood, their abdomens were visually examined to confirm their engorgement. The alimentary canal of each mosquito was then extracted by tugging on the last segment of the abdomen with fine forceps under Ringer solution. The Ringer solution consisted of (in mM): 150 NaCl, 3.4 KCl, 1.7 CaCl_2_, 1.8 NaHCO_3_, 1.0 MgCl_2_, 5 glucose, and 25 HEPES (pH 7.1). The Malpighian tubules were isolated from their attachment to the alimentary canal and immediately immersed in 50 µL of TRIzol Reagent (Life Technologies, Carlsbad, BA) in a sterile 1.5 ml microcentrifuge tube on ice. A total of 200 Malpighian tubules (from 40 females) were pooled for a given replicate.

Altogether, Malpighian tubules were isolated from 1) mosquitoes fed a blood meal at 3 different time points (3 h, 12 h, 24 h; 40 mosquitoes at each time point) and 2) mosquitoes not fed a blood meal at similar time points (40 mosquitoes each). A total of 3 biological replicates was obtained for each time point in both the BF and NBF groups, resulting in 18 sets of tubules for RNA isolation and cDNA library preparation (triplicates each of 3 h BF, 12 h BF, 24 h BF, 3 h NBF, 12 h NBF, and 24 h NBF).

### Isolation and purification of total RNA and assessment of its quantity and quality

Total RNA was extracted from each set of Malpighian tubules immediately after they were isolated from the mosquitoes using the method of Chomczynski and Sacchi [23]. The resulting RNA was treated with TURBO DNA-*free* (Life Technologies) to remove genomic DNA and then purified with a RNA Clean & Concentrator-5 kit (Zymo Research, Irvine, CA), according to the manufacturer's protocol.

The purified RNA was initially measured for quantity and quality using a NanoDrop 2000c Spectrophotometer (Thermo Fisher Scientific, Waltham, MA). Samples with a concentration <20 ng/µl or poor absorbance ratios (i.e., 260/280 value <1.6; 260/230 value <1.6 or >3.0) were discarded. RNA quality was further assessed using the Experion Automated Electrophoresis System (Bio-Rad, Hercules, CA). Only samples with RNA Quality indicator values of 7.5 or higher were used. The concentration of RNA was determined with a Qubit 2.0 Fluorometer (Life Technologies).

### Synthesis and sequencing of cDNA libraries

Total RNA (565 ng) was used to synthesize adaptor-indexed double-stranded cDNA libraries using the TruSeq DNA Sample Prep Kit V2, Set A and B (Illumina, San Diego, CA). The size chosen for libraries was ∼270 bp. The quality of the synthesized libraries was evaluated using the Agilent 2100 Bioanalyzer High Sensitive DNA Chip (Agilent Technologies, Santa Clara, CA) and the quantity determined using the Qubit 2.0 Fluorometer (Life Technologies).

The 18 resulting cDNA libraries were diluted to 18 nM and pooled to generate a multiplexed cDNA library (using 18 unique indexed adapters) of 36 fM. The pooled library was sequenced using the Illumina HiSeq 2000 platform at the Ohio State University Comprehensive Cancer Center (Columbus, OH). Demultiplexing was performed with CASAVA 1.8.2. FASTQ files were generated from the ‘basecall’ files. All single-end reads were submitted to the NCBI sequence read archive (accession number SRP034701). The sequencing of all 18 libraries generated over 232 million single-end raw reads (∼13 million reads per sample) (Table S1).

### Filtering data, alignment, and read counting

The MCIC-Galaxy pipeline was implemented for preprocessing, filtering, and data analysis [24]. The raw reads were first analyzed with the “FASTQC” tool (http://www.bioinformatics.babraham.ac.uk/projects/fastqc/) to assess quality. Adapters were removed using CUTADAPT [25] with an error rate of 0.1 and a minimum overlap length of 6. Reads were trimmed for length and quality using the “Trim the reads” tool, version 1.2.2, (Phred threshold score <20; read length <20 bp), discarding all miscalled bases, but not duplicates or polyA tails, because reads were aligned on a reference transcriptome. The number of reads retained after removing adapters, low quality, and short reads was >194 million, which is ∼84% of the total number of original 232 million reads.

After the preprocessing and filtering, reads were aligned to the following two reference transcriptomes using “Burrow-Wheeler Aligner”, version 1.2.3 [26]: 1) *A. aegypti* (www.vectorbase.org; AaegL1.4, v1.00; 18,769 sequences) [27] and 2) *A. albopictus* (www.ncbi.nlm.nih.gov;transcript shotgun archive accession numbers JO845359-JO913491, 68,413 sequences) [28]. The number of reads that aligned to unique and redundant transcripts in the reference transcriptome was determined for each sample using “Count Features” tool, version 0.91. The dataset was filtered to contain only transcripts with a minimum of five mapped reads for any two replicates in at least one treatment/time combination. Only the subset of transcripts meeting this criterion was used in subsequent analyses.

Considerably more reads mapped onto the *A. albopictus* transcriptome (∼9.6 million reads/sample; 75% reads mapped) compared to the *A. aegypti* transcriptome (∼2.4 million reads/sample; 18.5% reads mapped). However, the *A. albopictus* transcriptome assembly and annotation is incomplete and contains redundancies (i.e., there are several transcript identification numbers corresponding to the same transcript), which limited our ability to accurately identify and quantify specific individual transcripts of interest (e.g., aquaporins, NH_3_ detoxification enzymes). Moreover, mapping onto the *A. aegypti* transcriptome resulted in the detection of 9,813 non-redundant transcripts, which is within the range of ∼7,000–18,000 transcripts detected in RNA-seq studies of *A. aegypti* isolated tissues or whole animals [12], [17], [29], [30]. Thus, we use the *A. aegypti* transcriptome and its transcript nomenclature for our downstream analyses.

For quality assessment, “Count Features” output was implemented to examine the dispersion of the biological replicates (libraries per treatment). Counting reads were normalized calculating the RPKM (reads per kilobase per million reads). Pearson's correlation and Principal Component Analysis graphics were generated to assess the similarity of the replications for each condition.

### Transcriptome analysis

The read counts generated using “Count Features” output were submitted to the “DESeq” pipeline, version 1.0.0 [31], to identify transcripts with a significant differential expression between treatments/time points. This tool is based on the negative binomial distribution, with normalized libraries by size factor (developing an estimate effective library size) [31]. The comparisons made were a NBF treatment against BF treatments at three time points (3 h, 12 h and 24 h). All of the transcripts with a significant expression change were filtered by a FDR-adjusted *P* value threshold of 0.05, and their log_2_ fold-change value was recorded.

The DAVID v6.7 annotation clustering module [32] was used to classify differentially expressed transcripts into functional groups. Clustering analysis was carried out for the subset of transcripts that had showed sustained up- or down-regulation (i.e., at least two consecutive time points of differential expression). The DAVID is currently not compatible with *A. aegypti* transcript IDs. Thus, the differentially-expressed transcripts were first converted to *A. gambiae* transcript IDs using tBLASTx (E-value <10^−20^). Then, enrichment of GO and other annotation terms in candidate sub-lists were explored using the functional annotation clustering tool. This clustering method condenses the input transcript list into functionally related transcripts (annotation clusters), taking into account the similarity of their annotation profiles based on multiple annotation sources (e.g. GO terms and Interpro keywords).

The clusters are assigned an enrichment score, which represents the minus log-transformed geometric mean of the modified Fisher Exact (EASE) Scores within the cluster. Significantly enriched annotation clusters were defined as those containing a minimum enrichment score of 1.3, because the –log (0.05)  = 1.3. Thus, an enrichment score of >1.3 corresponds to a P<0.05. The enrichment score is based on the following parameters: Similarity Term Overlap  =  3; Similarity Threshold  =  0.7; Initial Group Membership  =  3; Final Group Membership  =  5; Multiple Linkage Threshold  =  0.3. Once the significantly-enriched functional clusters were identified, the *A. gambiae* transcript IDs within them were converted back to their respective *A. aegypti* transcript IDs.

We also performed a preliminary DAVID clustering analysis using reads mapped to the existing *A. albopictus* reference transcriptome, despite its incomplete assembly and annotation. Notably, there was a good correlation between the enriched functional clusters identified using this reference and those using the *A. aegypti* reference. For example, the thioredoxin, glutathione S-transferase, vitamin binding, cofactor metabolic process, oxidative phosphorylation/ATP synthesis, protein biosynthesis, glycoylsis, and ATPase activity clusters were enriched in both DAVID analyses. Thus, our decision to use the *A. aegypti* transcriptome, which allows for better identification and quantification of specific transcripts (see above), does not compromise our ability to identify enriched functional pathways.

## Results and Discussion

As explained in the Methods, Malpighian tubules were isolated from mosquitoes at 3 different time points after being fed a blood meal (3 h, 12 h, 24 h). In parallel, Malpighian tubules were isolated from mosquitoes that were not fed a blood meal at similar time points. A total of 3 biological replicates were obtained for each time point in both the blood fed (BF) and non-blood fed (NBF) groups.

**Figure 1:**
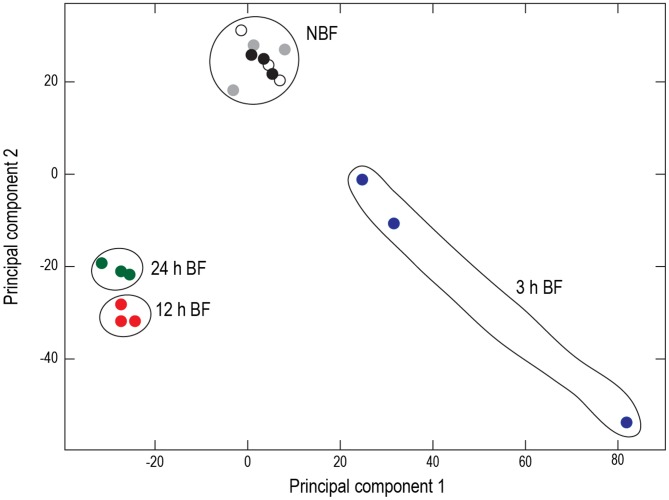
Principal component analysis of transcript expression in Malpighian tubules isolated from non-blood fed mosquitoes (NBF) at 3 h (gray circles), 12 h (white circles), and 24 h (black circles) and blood-fed (BF) mosquitoes at 3 h (blue circles), 12 h (red circles), and 24 h (green circles). Principal component 1 is time and principal component 2 is treatment (NBF, BF).

A principal component analysis (PCA) of the Malpighian-tubule transcriptomes derived from the 18 sequenced cDNA libraries (triplicates each of 3 h BF, 12 h BF, 24 h BF, 3 h NBF, 12 h NBF, and 24 h NBF) revealed distinct clustering of samples by treatment and time (Figure 1). Notably, all of the NBF samples clustered tightly with one another, regardless of time. Consistent with this observation, a GLM (general linear model) two-way ANOVA comparing the RPKM values among the NBF samples revealed no significant differences (*F*-value  =  0.731; Table S2), which indicates that time does not affect transcript accumulation in the NBF tubules. The PCA also showed that the BF samples clustered separately from their respective NBF controls and each other (Figure 1). Consistent with this observation, an ANOVA comparing the RPKM values among the BF samples revealed significant differences among all of the samples (*F*-value  =  0.017; Table S3), which indicates that time affects transcript accumulation in the BF tubules.

Below, we dissect the quantitative and qualitative differences among the NBF and BF samples in more detail. For simplicity and consistency, we selected the 24 h NBF samples as a universal control, because 1) an ANOVA found no significant transcript differences across all the time points for the NBF samples (see above) and 2) the 24 h NBF samples exhibited the lowest internal variation among the NBF samples (Figure 1). As described in the Methods, we used an assembled transcriptome for *A. aegypti* as a reference given its superior annotation and nominal redundancy compared to the available transcriptome for *A. albopictus*, which is still in its early stages of development.

### Differential expression in Malpighian tubules after blood feeding

DESeq was used to search for transcripts differentially expressed between the 24 h NBF control and the BF treatments at each time point. Using the *A. aegypti* transcriptome as a reference, a total of 1,857 non-redundant transcripts was found to be differentially expressed over all of the time points (∼10% of the *A. aegypti* transcriptome). Table 1 shows that the Malpighian tubules from the BF mosquitoes were characterized by progressive increases in differential expression throughout the time series. At each time point, the differentially expressed transcripts consist of similar numbers of up- and down-regulated transcripts.

**Table 1:** Total number of transcripts whose abundance is significantly affected by blood feeding at each time point compared to non-blood fed controls.

Treatment	3 h BF	12 h BF	24 h BF
Up-regulation	207	553	564
Down-regulation	192	482	669
**Total**	**399**	**1035**	**1233**

We next aimed to identify enriched, functional pathways within the differentially expressed transcripts using a DAVID functional clustering analysis [32], [33]. We focused our analysis on transcripts that exhibited ‘sustained’ changes in differential expression after blood feeding, which we defined as those significantly up- or down-regulated for at least two consecutive time periods. Based on the *A. aegypti* transcriptome, a total of 669 transcripts met our ‘sustained’ criterion, consisting of 340 up-regulated transcripts and 329 down-regulated transcripts. As shown in Table 2, the DAVID analysis revealed a significant enrichment (enrichment score > 1.3) of 1) nine functional groups among the sustained, up-regulated transcripts and 2) six functional groups among the sustained, down-regulated transcripts. The identities of the transcripts that comprise the functional groups of Table 2 and their respective heat maps of differential expression are shown in Figures S1–S15.

**Table 2:** DAVID functional clusters that are significantly enriched among the transcripts in Malpighian tubules exhibiting a sustained, up-regulation or down-regulation after blood feeding.

Functional cluster	Number of Transcripts	Enrichment Score	Functional cluster	Number of Transcripts	Enrichment Score
***Up-regulated***			***Down-regulated***		
Thioredoxin	17	4.05	Oxidative phosphorylation/ATP synthesis	40	5.28
Cofactor metabolic process	16	1.76	Protein biosynthesis	23	6.19
Tubulin, GTPase domain	15	1.61	Glycolysis	22	5.52
Amine biosynthetic process	11	1.86	ATPase activity	13	1.83
ATPase, AAA+ type	9	1.42	Translational elongation	6	3.07
Glutathione S-transferase	8	1.55	Sugar/inositol transporter	5	2.14
Vitamin binding	8	1.7			
Vitamin biosynthetic process	8	13.1			
Proteasome complex	7	3.05			

The transcripts that comprise each functional group are listed in Figures S1–S15.

Cursory interpretations of the changes to these broad functional groups and the transcripts within them suggests that blood feeding promotes the expression of transcripts associated with 1) antioxidants and detoxification, 2) proteolytic activity, 3) amino acid metabolism, and 4) cytoskeletal dynamics. On the other hand, blood feeding appears to suppress the expression of transcripts associated with 1) oxidative metabolism, 2) active transport, and 3) mRNA translation. Below, we discuss these interpretations in more detail with the caveat that transcript levels may not necessarily reflect protein abundance, biochemical activity, or physiological function. Thus, we consider our interpretations as the building of hypotheses that will require testing in future studies using functional genetic, biochemical, and physiological techniques.

### Down-regulation of transcripts associated with oxidative metabolism and active transport—Potential implications for diuretic fluid secretion

The mosquito Malpighian tubule epithelium is well-studied, because of its remarkable capacity for active transepithelial fluid secretion, which mediates the post-prandial diuresis. The vacuolar (V-type) H^+^-ATPase is the ultimate energizer of transepithelial fluid secretion in the epithelium [34]. This proton pump resides in the luminal brush border of principal cells where it is situated in close proximity to mitochondria that fuel the pump with ATP [35], [36]; the pump is a multisubunit protein consisting of two sectors: 1) a catalytic, cytosolic V_1_ sector and 2) a H^+^-translocating, membrane-bound V_0_ sector [37]. Inhibiting the pump or the production of ATP in the epithelium effectively inhibits fluid secretion [34], [38]. Thus, the enrichment of the ‘oxidative phosphorylation/ATP synthesis’, ‘ATPase activity’, ‘glycolysis’, and ‘sugar/inositol transporter’ functional clusters among the transcripts that exhibited a sustained down-regulation (Table 2) caught our attention.

**Figure 2:**
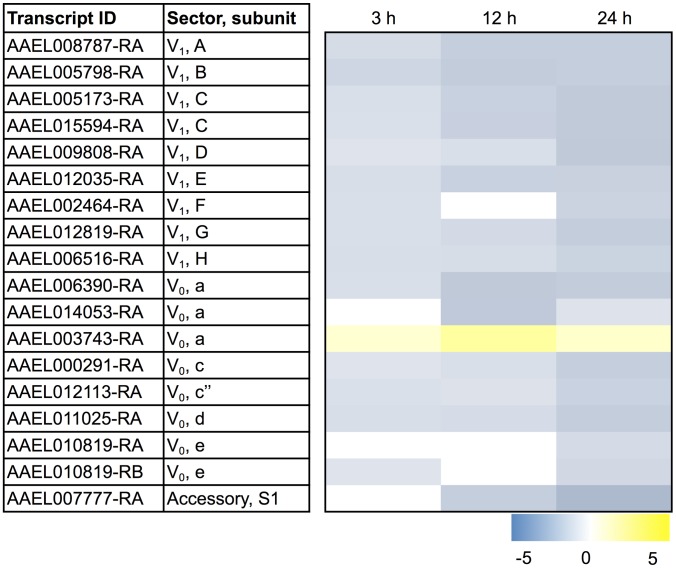
Differential expression after blood feeding of transcripts encoding subunits of the V-type H^+^-ATPase. Blue shading indicates significant down-regulation compared to NBF controls, whereas yellow shading indicates significant up-regulation compared to NBF controls. Lack of shading indicates no significant difference relative to NBF controls. Degree of shading is based on value of Log_2_ fold change as indicated by the scale below on the right.

Listed prominently among the down-regulated transcripts in the ‘oxidative phosphorylation/ATP synthesis’ (Figure S1) and ‘ATPase activity’ (Figure S2) functional clusters are those encoding subunits of the V-type H^+^-ATPase. Remarkably, as shown in Figure 2, fourteen transcripts encoding subunits of the V-type H^+^-ATPase exhibit a sustained down-regulation after blood feeding, while only one shows a sustained up-regulation (AAEL003743-RA). Furthermore, a manual search of all the differentially-expressed transcripts (including those not considered ‘sustained’) revealed three other transcripts associated with the V-type H^+^-ATPase that are down-regulated transiently at one or two non-consecutive time points (i.e., AAEL002464-RA, AAEL010819-RA, AAEL010819-RB in Figure 2). Among all of these down-regulated transcripts, nine encode subunits of the V_1_ sector of the V-type H^+^-ATPase, seven encode subunits of the V_0_ sector, and one encodes an accessory protein (Figure 2). The only up-regulated transcript encodes subunit ‘a’ of the V_0_ sector (a.k.a. vha100-1).

The above changes to transcripts of the V-type H^+^-ATPase in the Malpighian tubules of *A. albopictus* contrast with those previously reported in the midgut of *A. aegypti* after blood feeding [19]. In our study, nearly all subunits of the V-type H^+^-ATPase exhibited a down-regulation in Malpighian tubules after blood feeding (Figure 2), whereas Sanders et al. found that transcripts encoding V-type H^+^-ATPase subunits exhibited an up-regulation in the midgut of *A. aegypti* at 12 h and 24 h after blood feeding [19]. These contrasting results suggest that the regulation of V-type H^+^-ATPase expression in response to blood feeding is tissue dependent in mosquitoes.

Notable among the transcripts that exhibited a sustained down-regulation after blood feeding in the ‘oxidative phosphorylation/ATP synthesis’ (Figure S1) and ‘glycolysis’ (Figure S3) functional clusters are those encoding enzymes associated with glycolysis, the citric acid cycle, and ATP synthesis, such as phosphofructokinase, pyruvate kinase, enolase, 2-oxoglutarate dehydrogenase, glycerol-3-phosphate dehydrogenase, and acetyl-CoA synthetase. In addition, within the ‘oxidative phosphorylation/ATP synthesis’ (Figure S1) and ‘sugar/inositol transporter’ (Figure S4) functional clusters are transcripts encoding putative SLC2-like sugar transporters, which import glucose into cells for use as a fuel to generate ATP. Thus, in Malpighian tubules, blood-feeding leads to a decrease in the abundance of transcripts encoding enzymes associated with the catabolism of glucose and synthesis of ATP, which is consistent with the aforementioned decrease in abundance of transcripts encoding subunits of the V-type H^+^-ATPase subunits.

Also notable among the transcripts listed in the ‘oxidative phosphorylation/ATP synthesis’ (Figure S1) functional cluster are those encoding ion transport mechanisms that are known or hypothesized to play a role in the transepithelial secretion of ions by mosquito Malpighian tubules. We discuss these mechanisms below.

#### Mechanisms of K^+^ uptake

**Figure 3:**
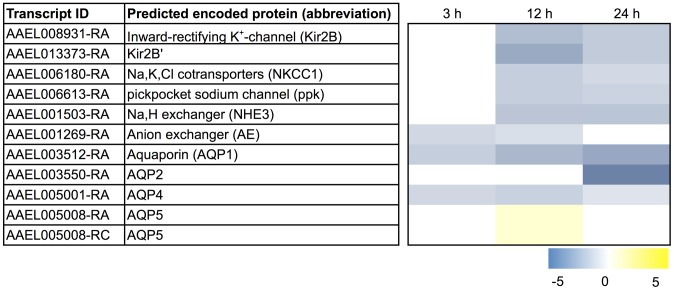
Differential expression after blood feeding of transcripts encoding known and putative ion and water transport mechanisms. Blue shading indicates significant down-regulation compared to NBF controls, whereas yellow shading indicates significant up-regulation compared to NBF controls. Lack of shading indicates no significant difference relative to NBF controls. Degree of shading is based on value of Log_2_ fold change as indicated on the right.

Both barium-sensitive inward-rectifying K^+^ (Kir) channels and bumetanide-sensitive Na,K,2Cl-cotransporters (NKCCs) are considered the major mechanisms for the uptake of K^+^ from the hemolymph across the basolateral membranes of mosquito Malpighian tubules [39], [40], [41], where they presumably localize to principal cells. As shown in Figure 3, blood-feeding elicits a sustained down-regulation of two transcripts encoding Kir channel subunits (AAEL008931-RA, AAEL013373-RA) and one transcript encoding a putative NKCC (AAEL006180-RA). We recently cloned and characterized AAEL008931-RA (Kir2B) from the Malpighian tubules of *A. aegypti* and have confirmed that it encodes a barium-sensitive Kir channel [41]. The NKCC is an ortholog of *Drosophila melanogaster* Ncc69, which has been shown to a play a key role in fluid and K^+^ secretion in fruit fly Malpighian tubules [42]. These changes in Kir and NKCC transcript abundance suggest that blood-feeding leads to a decrease in the capacity of the tubules to uptake K^+^ across the basolateral membrane, which is expected to reduce the diuretic capacity of the tubules.

#### Mechanisms of Na^+^ uptake

A cAMP-induced, amiloride-sensitive Na^+^-conductance [40], [43], [44] and an amiloride-insensitive Na/H exchanger (NHE3) [45] are considered important mechanisms for the uptake of Na^+^ across the basolateral membranes of mosquito Malpighian tubules, where they are hypothesized and known, respectively, to localize to principal cells. Although the molecular identity of the Na^+^-conductance is unknown, one transcript encoding a putative ‘pickpocket’ Na^+^ channel exhibits a sustained down-regulation after blood feeding (Figure 3). Pickpocket genes contribute to 1) transepithelial movements of fluid in the tracheal tubes [46] and 2) osmosensation in the nervous system of *Drosophila* [47]. Thus, it is reasonable to hypothesize a potential role of pickpocket in fluid secretion by mosquito Malpighian tubules. Moreover, a transcript encoding NHE3 shows a similar pattern of down-regulation as the pickpocket transcript after blood feeding (Figure 3). These changes in pickpocket and NHE3 abundance suggest that blood-feeding leads to a decrease in the capacity of the tubules to uptake Na^+^ across the basolateral membrane, which is expected to reduce the diuretic capacity of the tubules.

#### Mechanisms of HCO_3_^-^ transport

The basolateral membranes of mosquito Malpighian tubules possess a DIDS-sensitive Cl/HCO_3_ anion exchanger (AE), which localizes to stellate cells [48], [49]. We have shown that this AE plays a key role in diuretic fluid secretion in isolated Malpighian tubules of *A. aegypti* by presumably regulating the oxidative metabolism of principal cells [7], [48]. The AE may also contribute to the transepithelial secretion of Cl^-^ by stellate cells [7]. Notably, after blood feeding, a transcript encoding the AE (AAEL001269-RA) is down-regulated (Figure 3). This change suggests that blood feeding leads to a decrease in the capacity of stellate cells to regulate the oxidative metabolism of principal cells and to transport Cl^−^, which is expected to decrease the diuretic capacity of the tubules.

#### Mechanisms of water transport

Given the changes to the above ion transport mechanisms, we also manually searched for differentially-expressed transcripts encoding aquaporin water channels (AQPs). Although AQP transcripts were not part of the enriched functional clusters identified by the DAVID analysis, the Malpighian tubules of *A. aegypti* express at least three AQPs (AQP1, AQP4, AQP5) that play an important role in the post-prandial diuresis and exhibit differential expression after blood feeding [22]. Furthermore, AQP1 immunoreactivity is expressed in stellate cells and some principal cells in the Malpighian tubules of *A. gambiae* [50], [51].

As shown in Figure 3, transcripts encoding AQP1 and AQP4 exhibit a sustained down-regulation after blood feeding and a transcript encoding AQP2 is down-regulated by 24 h. In contrast, two transcripts encoding AQP5 isoforms are up-regulated, but only transiently at 12 h (Figure 3). Surprisingly, the changes in AQP expression we observed in *A. albopictus* Malpighian tubules contrast with those found in the Malpighian tubules of *A. aegypti* by Drake and colleagues [22]. In particular, at 3 h after a blood meal, AQP1, AQP2, and AQP4 each exhibited transient increases of expression in the Malpighian tubules of *A. aegypti* [22], whereas we observed decreases of all three within 24 h in *A. albopictus*. Moreover, in *A. albopictus*, we observed a transient increase of AQP5 at 12 h (Figure 3), whereas in *A. aegypti*, AQP5 expression was increased in the tubules at 3 h, 12 h, and 24 h after blood feeding [22]. These contrasts may point to species-specific differences in how the AQPs of mosquito Malpighian tubules respond to the physiological stresses encountered during blood feeding.

The consensus of the above findings in Figures S1–S4 and Figures 2–3 suggests that as early as 3 h after a blood meal, the Malpighian tubule epithelium initiates a host of molecular changes that are expected to reduce its capacity for 1) generating transepithelial electrochemical gradients by the V-type H^+^-ATPase, and 2) mediating the transepithelial transport of K^+^, Na^+^, Cl^−^ and water. Thus, during the chronic processing of blood meals (24–48 h post blood meal) the capacity of the Malpighian tubules to mediate active, transepithelial fluid secretion (i.e., diuresis) may decrease. Such a reduction in diuretic capacity would make physiological sense given that the major stresses to hemolymph salt and water homeostasis are alleviated within 2 h after blood feeding [8].

### Down-regulation of transcripts associated with the translation of mRNA

The other functional clusters enriched among the transcripts that exhibited a sustained down-regulation are related to the translation of mRNA (i.e., protein biosynthesis and translational elongation; Table 2). These transcripts consist primarily of ribosomal protein subunits and translation/elongation factors that exhibit a down-regulation at 12 h and 24 h after a blood meal (Figure S5 and Figure S6), which suggests that most newly-translated proteins in the tubules in response to blood feeding are synthesized within 12 h. Furthermore, the data suggest that during the chronic processing of blood meals (24–48 h after blood feeding) the capacity of the tubules for de novo protein synthesis may decrease. These observations in the Malpighian tubules of *A. albopictus* are similar to results of previous studies in the midgut and fat body of *A. aegypti*, where blood feeding led to a down-regulation of transcripts encoding ribosomal protein subunits and/or translation factors within 12 h to 24 h after a blood meal [17], [19], [52].

### Up-regulation of transcripts associated with antioxidants and detoxification—Implications for heme metabolism/detoxification and metabolite excretion

Hemoglobin is the most abundant protein in mammalian blood, and its digestion leads to the production of heme, a highly toxic metabolite that causes cell and tissue damage via oxidative stress and/or the disruption of plasma membranes [53]. Mosquitoes utilize a variety of mechanisms to detoxify heme and limit its absorption into the hemolymph. The first line of defense is found in the midgut, which secretes a peritrophic matrix that encapsulates the ingested blood cells and may sequester more than half of the amount of heme in a typical blood meal [54], [55]. Additional mechanisms for heme detoxification include the 1) enzymatic degradation of heme by heme oxygenase (HO) to produce biliverdin, Fe^2+^, and carbon monoxide [56], 2) chelation of heme by xanthurenic acid (XA), which is a product of the kynurenine pathway of tryptophan catabolism [57], [58], and 3) binding and/or catabolism of heme by glutathione S-transferases (GSTs) [59], [60]. Furthermore, protection against heme-induced, free-radical damage in mosquitoes can be mediated by: 1) antioxidant enzymes, such as glutathione peroxidase (GP), thioredoxin peroxidase (THP), thioredoxin reductase (THR), superoxide dismutase (SOD), and catalase (CAT); 2) antioxidant proteins, such as thioredoxin (TH); and 3) small molecule antioxidants, such as glutathione and uric acid [53].

Thus, we were intrigued by the enrichment of the ‘thioredoxin’ and ‘glutathione S-transferase’ functional clusters among the transcripts that exhibited a sustained up-regulation after blood feeding (Table 2; Figures S7–S8). Furthermore, we noticed that the ‘cofactor metabolic process’ (Figure S9) and ‘vitamin biosynthetic process’ (Figure S10) functional clusters contained several transcripts associated with putative antioxidant and detoxification mechanisms, and that the ‘ATPase/AAA+ type’ (Figure S11) functional cluster contained several transcripts encoding putative ATP-binding cassette (ABC) transporters, which play key roles in insect metabolite/xenobiotic excretion [61]. Below, we discuss the up-regulation of transcripts after a blood meal within the aforementioned functional clusters in the context of 1) the prevention of heme-induced oxidative cell and tissue damage and 2) the detoxification/excretion of heme and heme-related metabolites.

#### Heme detoxification and antioxidant production mechanisms

**Figure 4:**
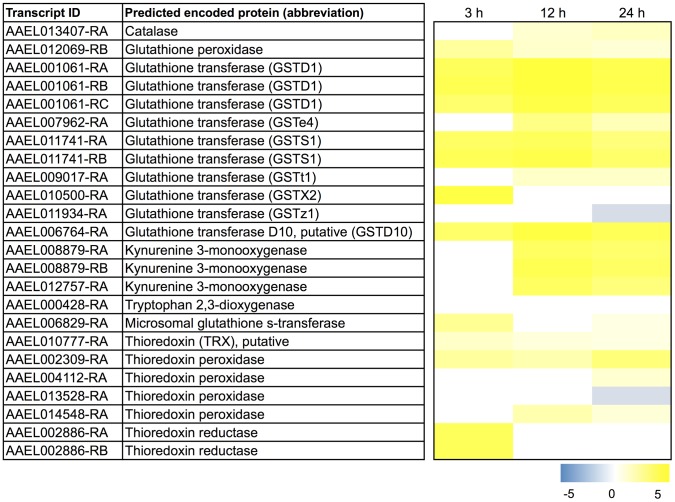
Differential expression after blood feeding of transcripts associated with heme detoxification and antioxidant mechanisms. Blue shading indicates significant down-regulation compared to NBF controls, whereas yellow shading indicates significant up-regulation compared to NBF controls. Lack of shading indicates no significant difference relative to NBF controls. Degree of shading is based on value of Log_2_ fold change as indicated by the scale below on the right.

As shown in Figure 4, we find a remarkable, sustained up-regulation after blood feeding of several transcripts encoding putative heme-detoxification mechanisms, including 4 transcripts that encode enzymes associated with XA production (kynurenine 3-monoxygenases and a tryptophan dioxygenase) and 8 transcripts encoding GSTs (Delta, Sigma, Epsilon, and Rho classes). A manual search for other GSTs among the transcripts that were differentially expressed (non-sustained) revealed 3 others: 1) a putative microsomal GST (AAEL006829-RA), which was up-regulated, albeit transiently, at 3 h and 24 h after blood feeding, 2) the heme-binding GSTX2 (AAEL010500-RA), which was up-regulated transiently at 3 h, and 3) a Zeta class GST (AAEL011934-RA), which was down-regulated by 24 h after blood feeding (Figure 4). Interestingly, two of the up-regulated GST transcripts (AAEL007962-RA, GSTe4; AAEL010500-RA, GSTX2) were also found to be up-regulated in pyrethroid-resistant lines of *A. aegypti* [62], [63], which suggests putative roles of these GSTs in both heme and insecticide detoxification in mosquitoes.

Transcripts encoding putative antioxidant mechanisms for protection against heme-induced oxidative stress are also in general up-regulated in Malpighian tubules after blood feeding. Namely, transcripts encoding CAT, GP, TH, THP, and THR exhibited a sustained up-regulation after a blood meal (Figure 4). A manual search for additional differentially-expressed transcripts (non-sustained) encoding THR revealed two others (AAEL002886-RA,-RB), which were transiently up-regulated at 3 h (Figure 4); a similar search for transcripts encoding THPs found one (AAEL004112-RA) that is up-regulated by 24 h and another (AAEL013528-RA) that is down-regulated by 24 h (Figure 4). Similar patterns in the expression of CAT and THR have been observed in the midgut of *A. aegypti* after a blood meal [19].

The consensus of the above results suggests that the molecular capacity of the Malpighian tubules to detoxify heme and protect against heme-induced free radical damage increases after mosquitoes ingest a blood meal. Thus, the data may point towards a putative new role of the tubule epithelium after a blood meal in the detoxification of heme that enters the hemolymph.

#### Heme and heme-metabolite excretion

In addition to direct heme detoxification, the up-regulation of GSTs can be interpreted as serving a potential role in improving the clearance of heme metabolites, such as biliverdin (derived from HO activity), from the hemolymph. Mammalian GSTs possess binding-sites for various organic molecules, including biliverdin, bilirubin, and even heme [64], [65], [66]. In the mammalian liver, the binding of bilirubin by intracellular GSTs provides a favorable diffusional gradient for bilirubin uptake by liver cells [65]. Moreover, GSTs are known to conjugate reduced glutathione onto large organic molecules thereby increasing their solubility, decreasing their toxicity, and enhancing their transport by ABC transporters [67]. Thus, it is reasonable to hypothesize that one or more of the up-regulated GSTs in Malpighian tubules may contribute towards 1) maintaining a favorable chemical gradient for the uptake of heme and/or biliverdin from the hemolymph, and/or 2) chemically modifying heme and/or biliverdin to improve their excretion.

**Figure 5:**
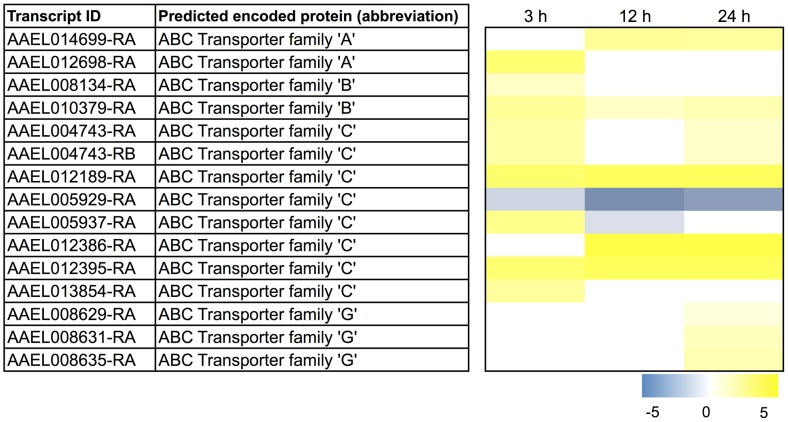
Differential expression after blood feeding of transcripts encoding putative ABC transporters. Blue shading indicates significant down-regulation compared to NBF controls, whereas yellow shading indicates significant up-regulation compared to NBF controls. Lack of shading indicates no significant difference relative to NBF controls. Degree of shading is based on value of Log_2_ fold change as indicated by the scale below on the right.

In regards to the potential excretion of heme and heme-derived metabolites by Malpighian tubules, putative ABC transporters are among those in the ‘ATPase/AAA+ type’ functional cluster (Figure S11). In general, ABC transporters are promiscuous in their transport substrates, which include heme, bilirubin, glutathione-conjugated organic compounds, uric acid, xenobiotics (including insecticides), ions (e.g., cytstic-fibrosis transmembrane regulator), and lipids [68], [69], [70], [71], [72]. As shown in Figure 5, five transcripts encoding ABC transporters (of the ‘A’, ‘B’, and ‘C’ types) exhibit a sustained up-regulation after blood feeding. Furthermore, a manual search of all the differentially-expressed transcripts (non-sustained) revealed eight others (of the ‘A’, ‘B’, ‘C’, and ‘G’ types) that exhibited a transient up-regulation at one or two discontinuous time points (Figure 5). Only one transcript (AAEL005929-RA) exhibited a down-regulation and another (AAEL005937-RA) exhibited an up-regulation at 3 h followed by a down-regulation at 12 h. Thus, there is a general up-regulation of transcripts encoding ABC transporters, which suggests that blood feeding leads to an increased capacity of the tubule epithelium for the excretion of metabolites and xenobiotics derived from the blood meal.

### Up-regulation of transcripts associated with proteasomes—Implications for proteolytic activity

Another functional cluster enriched among the transcripts that exhibited a sustained up-regulation is the ‘proteasome complex’ (Table 2), which is related to the degradation of protein. These transcripts consist entirely of those encoding proteasome or protease regulatory subunits and most exhibit an up-regulation at 3 h and 12 h after a blood meal (Figure S12), which suggests that the tubules enhance their molecular capacity for proteolytic activity early after a blood meal. Enhanced proteolytic activity within the tubule epithelium may lead to the degradation of 1) proteins encoded by the transcripts that are down-regulated after blood feeding, and/or 2) proteins that may experience oxidative damage from heme. An increase of proteasome activity would also be expected to result in an increase of free amino-acids available for the synthesis of new proteins [73]—perhaps those associated with the ‘thioredoxin’ and ‘glutathione S-transferase’ functional clusters mentioned above.

### Up-regulation of transcripts associated with amino acid metabolism—Potential insights into ammonia detoxification

Consistent with the enrichment of the ‘proteasome complex’ functional cluster among up-regulated transcripts, which is expected to increase the abundance of free amino acids (see above), there is a corresponding enrichment in the ‘amine biosynthetic process’ cluster (Table 2). The transcripts within this cluster consist primarily of those encoding enzymes associated with amino-acid catabolism and/or biosynthesis, such as cysteine dioxygenase, 2-amino-3-ketobutyrate coenzyme A ligase, glutamine synthetase, phosphoserine phosphatase, ornithine decarboxylase, and phosphoserine aminotransferase (Figure S13). Similar (as well as redundant) transcripts are also found in the ‘vitamin binding’ functional cluster, such as alanine-glyoxylate aminotransferase and alanine aminotransferase (Figure S14). Notably, the up-regulation of these transcripts occurs at 12 h and 24 h, appearing to follow the up-regulation of transcripts associated with the proteasome (Figure S12). Thus, the potential increase in the availability of amino acids derived from proteasome activity in the tubules may be followed by an increased molecular capacity to breakdown and/or convert amino acids into other products.

**Figure 6:**
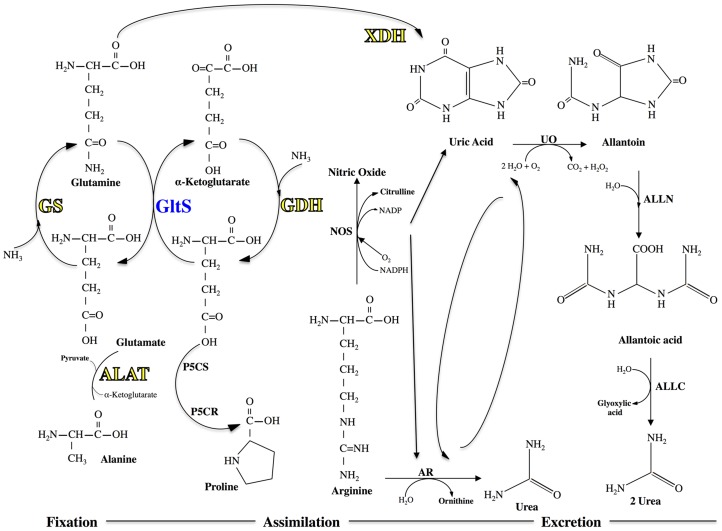
Biochemical pathways for ammonia detoxification in mosquitoes. Redrawn from Isoe and Scaraffia [9]. Yellow and blue text indicates an enzyme encoded by a transcript that is up-regulated and down-regulated, respectively, after blood feeding.

The aforementioned up-regulation of glutamine synthetase and alanine aminotransferase drew our attention to a potential role of the Malpighian tubules in the handling of ammonia. As mosquitoes metabolize a protein-rich blood meal, they face a potentially toxic accumulation of ammonia in their hemolymph and tissues from the catabolism of proteins and amino acids in the blood meal. Glutamine synthetase and alanine aminotransferase play prominent roles in detoxifying the ammonia (see below and Figure 6).

In brief, to prevent the build-up of toxic ammonia, mosquitoes have a remarkable capacity to fix and assimilate it into free amino acids (e.g., alanine, proline, glutamine) via a series of biochemical reactions catalyzed by enzymes, such as glutamine synthetase (GS), glutamate dehydrogenase (GDH), glutamate synthase (GltS), alanine aminotransferase (ALAT), and pyrrolidine-5-carboxylate synthase (P5CS) and reductase (P5CR) (Figure 6) [9], [74], [75], [76]. Moreover, glutamine can serve as a substrate for the production of uric acid through a pathway that includes xanthine dehydrogenase (XDH) among other enzymes [9], [76]. Once uric acid is produced, it can be excreted directly or further converted into allantoin, allantoic acid, and urea through a series of biochemical reactions catalyzed by urate oxidase (UO), allantoinase (ALLN), and allantoicase (AALC), respectively (Figure 6) [9], [76].

**Figure 7:**
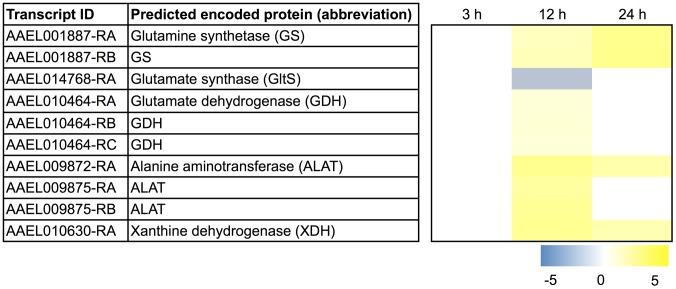
Differential expression after blood feeding of transcripts associated with ammonia detoxification. Blue shading indicates significant down-regulation compared to NBF controls, whereas yellow shading indicates significant up-regulation compared to NBF controls. Lack of shading indicates no significant difference relative to NBF controls. Degree of shading is based on value of Log_2_ fold change as indicated by the scale below on the right.

Figure 7 shows the transcripts associated with this pathway in Malpighian tubules that are differentially expressed after blood feeding. Namely, two transcripts encoding GS, one transcript encoding ALAT, and one transcript encoding XDH exhibit a sustained up-regulation after blood feeding. Three transcripts encoding a GDH and two transcripts encoding ALAT are each transiently up-regulated at 12 h, whereas one transcript encoding GltS is transiently down-regulated at 12 h (Figure 7). The up-regulation of these transcripts occurs at 12 h and/or 24 h after blood feeding, which coincides with the putative availability of amino acids from increased proteasome activity in the tubule (see above), as well as a period of intense protein digestion of the blood meal in the midgut [10], [11].

Interpreting these molecular findings in the context of the ammonia detoxification pathway suggests that the Malpighian tubules detoxify ammonia by converting it to glutamate via GDH and/or glutamine via GS (Figure 6). Given the up-regulation of ALAT, it is reasonable to propose that any newly-formed glutamate is converted into alanine (Figure 6). This putative handling of ammonia is similar to that reported for the midgut of *A. aegypti*, which fixes and assimilates ammonia into glutamine and alanine, as opposed to the fat body which fixes and assimilates ammonia into glutamine and proline [77]. The fate of the alanine in Malpighian tubules is unknown, but it is possible that it is shuttled to the fat body for conversion into proline, which can then be shuttled to the flight muscle for use as an energy source [78].

The fate of glutamine in Malpighian tubules is also unknown, but it is possible that it is converted into uric acid for excretion, as indicated by 1) the sustained up-regulation of XDH, and 2) the transient down-regulation of GltS, after blood feeding (Figure 7). Since transcripts encoding UO, ALLN, and ALLC were not differentially expressed after blood feeding (data not shown), the uric acid may be directly secreted by the tubules for excretion (perhaps by an ABC transporter in Figure 5), or it may be retained by the epithelium for use as an antioxidant to combat potential oxidative damage due to heme. Consistent with the former notion, uric acid is excreted by mosquitoes after a blood meal [79]. Likewise, uric acid is excreted by tsetse flies and reduviid bugs following a blood meal [80], [81], [82].

### Up-regulation of transcripts associated with cytoskeletal dynamics

The remaining functional cluster enriched among the transcripts that exhibited a sustained up-regulation is related to cytoskeletal dynamics (i.e., ‘tubulin, GTPase domain’) (Table 2). The transcripts in this functional cluster consist primarily of those encoding components of microtubules, such as tubulin chains (alpha and beta), dynein light chains, and microtubule-associated proteins (Figure S15). Another related transcript populating this cluster is one encoding a putative Rab GTPase (AAEL006091-RA); Rab GTPases play important roles in regulating microtubule-mediated trafficking of vesicular cargo [83]. These changes suggest that blood feeding leads to a more dynamic microtubule-based cytoskeleton, which may be associated with the intracellular trafficking of vesicles and/or organelles. At least one study has found that the actin cytoskeleton of principal cells plays a key role in modulating diuretic fluid secretion in *A. aegypti* Malpighian tubules [84].

Putative changes in the microtubule-based cytoskeleton would also be consistent with a potential functional transition of the epithelium after blood feeding. For example, if the capacity of the tubule for diuresis indeed decreases, as expected by the down-regulation of transcripts associated with the V-type H^+^-ATPase and other ion/water transport mechanisms (Figures 2–3), then enhanced vesicular trafficking may play a key role in the endocytosis and degradation of these membrane-bound proteins. Likewise, if the capacity of the tubules for detoxification and metabolite excretion indeed increases, as expected by the up-regulation of transcripts associated with heme and ammonia detoxification/excretion (Figures 4–6), then the cytoskeletal dynamics may facilitate the movements of newly synthesized membrane-bound transporters that mediate the excretion of metabolites, such as ABC transporters.

It is also possible that a more dynamic microtubule cytoskeleton would facilitate the movements of organelles within the epithelial cells. For example, retraction of mitochrondria from the apical microvilli in principal cells is associated with a decrease of fluid secretion in Malpighian tubules isolated from pupal stages of mosquitoes [85]. Thus, similar microtubule-mediated movements of mitochondria may occur during the chronic processing of blood meals (24–48 h after blood feeding) to further contribute to a putative decreased capacity for diuresis.

### Conclusions and significance

**Figure 8:**
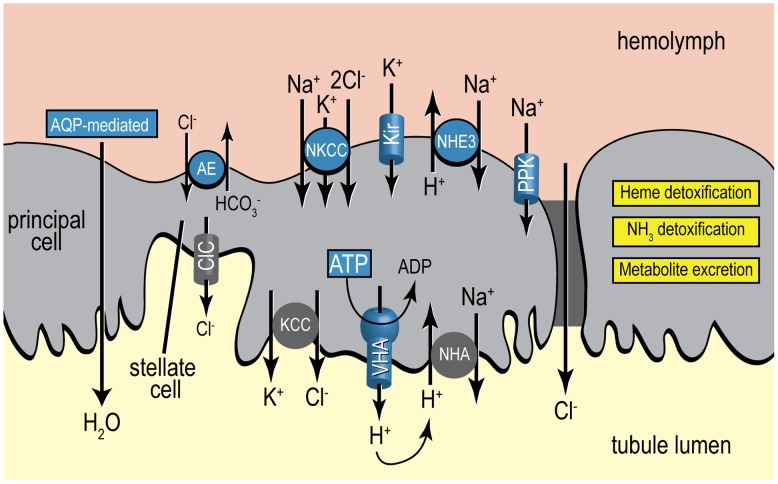
Summary of the molecular changes identified in the Malpighian tubule epithelium after blood feeding in the present study, with an emphasis on the mechanisms of transepithelial fluid secretion. Blue-shaded items represent putative mechanisms or pathways that are down-regulated after blood feeding, whereas yellow-shaded text boxes represent putative pathways or functions that are up-regulated after blood feeding. See text for details. Gray-shaded items are ion transport mechanisms not examined in the present study. AE, anion exchanger [48]; AQP, aquaporin [22]; ATP, adenosine triphosphate [38]; ADP adenosine diphosphate; ClC, chloride channel [86]; Kir, inward-rectifying potassium channel [41]; KCC, potassium-chloride cotransporter [87]; PPK, pickpocket sodium channel (hypothesized, present study); NHA, sodium-hydrogen antiporter [88]; NHE, sodium-hydrogen exchanger [45]; NKCC, sodium-potassium-chloride cotransporter [89]; VHA, V-type H^+^-ATPase [35].

The present study provides the first transcriptomic analysis of the Malpighian tubules of a mosquito after a blood meal, and is also the first to be conducted in *A. albopictus* after blood feeding. The results reveal molecular changes in transcript accumulation in the tubule epithelium within the first 24 h after a blood meal that suggest a remarkable functional transition of the epithelium from one dedicated to electrolyte and fluid excretion to one dedicated to detoxification and metabolite processing (Figure 8). Moreover, the results uncover new putative roles of the Malpighian tubules in the chronic processing of blood meals after the post-prandial diuresis ends ∼2 h after a blood meal [8]. Thus, the tubule epithelium may represent an even more valuable target for the development of novel insecticides than has been previously appreciated. The next important step to complete is to validate the hypothesized functional transition of the epithelium after a blood meal using biochemical and physiological approaches.

## Supporting Information

Figure S1 Lists of transcripts that exhibited a sustained down-regulation after blood feeding and were associated with the ‘oxidative phosphorylation/ATP synthesis’ DAVID functional cluster (see Table 2). Blue shading indicates significant down-regulation compared to NBF controls. Lack of shading indicates no significant difference relative to NBF controls. Degree of shading is based on value of Log_2_ fold change as indicated by the scale below on the right.
(TIF)

Figure S2 Lists of transcripts that exhibited a sustained down-regulation after blood feeding and were associated with the ‘ATPase activity’ DAVID functional cluster (see Table 2). Blue shading indicates significant down-regulation compared to NBF controls. Lack of shading indicates no significant difference relative to NBF controls. Degree of shading is based on value of Log_2_ fold change as indicated by the scale below on the right.
(TIF)

Figure S3 Lists of transcripts that exhibited a sustained down-regulation after blood feeding and were associated with the ‘glycolysis’ DAVID functional cluster (see Table 2). Blue shading indicates significant down-regulation compared to NBF controls. Lack of shading indicates no significant difference relative to NBF controls. Degree of shading is based on value of Log_2_ fold change as indicated by the scale below on the right.
(TIF)

Figure S4 Lists of transcripts that exhibited a sustained down-regulation after blood feeding and were associated with the ‘sugar/inositol transporter’ DAVID functional cluster (see Table 2). Blue shading indicates significant down-regulation compared to NBF controls. Lack of shading indicates no significant difference relative to NBF controls. Degree of shading is based on value of Log_2_ fold change as indicated by the scale below on the right.
(TIF)

Figure S5 Lists of transcripts that exhibited a sustained down-regulation after blood feeding and were associated with the ‘protein biosynthesis’ DAVID functional cluster (see Table 2). Blue shading indicates significant down-regulation compared to NBF controls. Lack of shading indicates no significant difference relative to NBF controls. Degree of shading is based on value of Log_2_ fold change as indicated by the scale below on the right.
(TIF)

Figure S6 Lists of transcripts that exhibited a sustained down-regulation after blood feeding and were associated with the ‘translational elongation’ DAVID functional cluster (see Table 2). Blue shading indicates significant down-regulation compared to NBF controls. Lack of shading indicates no significant difference relative to NBF controls. Degree of shading is based on value of Log_2_ fold change as indicated by the scale below on the right.
(TIF)

Figure S7 Lists of transcripts that exhibited a sustained up-regulation after blood feeding and were associated with the ‘thioredoxin’ DAVID functional cluster (see Table 2). Yellow shading indicates significant up-regulation compared to NBF controls. Lack of shading indicates no significant difference relative to NBF controls. Degree of shading is based on value of Log_2_ fold change as indicated by the scale below on the right.
(TIF)

Figure S8 Lists of transcripts that exhibited a sustained up-regulation after blood feeding and were associated with the ‘glutathione S-transferase’ DAVID functional cluster (see Table 2). Yellow shading indicates significant up-regulation compared to NBF controls. Lack of shading indicates no significant difference relative to NBF controls. Degree of shading is based on value of Log_2_ fold change as indicated by the scale below on the right.
(TIF)

Figure S9 Lists of transcripts that exhibited a sustained up-regulation after blood feeding and were associated with the ‘cofactor metabolic process’ DAVID functional cluster (see Table 2). Yellow shading indicates significant up-regulation compared to NBF controls. Lack of shading indicates no significant difference relative to NBF controls. Degree of shading is based on value of Log_2_ fold change as indicated by the scale below on the right.
(TIF)

Figure S10 Lists of transcripts that exhibited a sustained up-regulation after blood feeding and were associated with the ‘vitamin biosynthetic process’ DAVID functional cluster (see Table 2). Yellow shading indicates significant up-regulation compared to NBF controls. Lack of shading indicates no significant difference relative to NBF controls. Degree of shading is based on value of Log_2_ fold change as indicated by the scale below on the right.
(TIF)

Figure S11 Lists of transcripts that exhibited a sustained up-regulation after blood feeding and were associated with the ‘ATPase, AAA+ type’ DAVID functional cluster (see Table 2). Yellow shading indicates significant up-regulation compared to NBF controls. Lack of shading indicates no significant difference relative to NBF controls. Degree of shading is based on value of Log_2_ fold change as indicated by the scale below on the right.
(TIF)

Figure S12 Lists of transcripts that exhibited a sustained up-regulation after blood feeding and were associated with the ‘proteasome complex’ DAVID functional cluster (see Table 2). Yellow shading indicates significant up-regulation compared to NBF controls. Lack of shading indicates no significant difference relative to NBF controls. Degree of shading is based on value of Log_2_ fold change as indicated by the scale below on the right.
(TIF)

Figure S13 Lists of transcripts that exhibited a sustained up-regulation after blood feeding and were associated with the ‘amine biosynthetic process’ DAVID functional cluster (see Table 2). Yellow shading indicates significant up-regulation compared to NBF controls. Lack of shading indicates no significant difference relative to NBF controls. Degree of shading is based on value of Log_2_ fold change as indicated by the scale below on the right.
(TIF)

Figure S14 Lists of transcripts that exhibited a sustained up-regulation after blood feeding and were associated with the ‘vitamin binding’ DAVID functional cluster (see Table 2). Yellow shading indicates significant up-regulation compared to NBF controls. Lack of shading indicates no significant difference relative to NBF controls. Degree of shading is based on value of Log_2_ fold change as indicated by the scale below on the right.
(TIF)

Figure S15 Lists of transcripts that exhibited a sustained up-regulation after blood feeding and were associated with the ‘tubulin, GTPase domain’ DAVID functional cluster (see Table 2). Yellow shading indicates significant up-regulation compared to NBF controls. Lack of shading indicates no significant difference relative to NBF controls. Degree of shading is based on value of Log_2_ fold change as indicated by the scale below on the right.
(TIF)

Table S1 Number of ‘raw reads’ and ‘reads mapped’ for the Malpighian tubule cDNA libraries that were sequenced using RNA-Seq.
(DOCX)

Table S2 ANOVA of non-blood fed libraries across time points. Number of reads per transcript per library was used as the independent variable.
(DOCX)

Table S3 ANOVA of blood fed libraries across time points. Number of reads per transcript per library was used as the independent variable.
(DOCX)
