# Supplementary figures and images for: Transcriptomic Evidence for a Dramatic Functional Transition of the Malpighian Tubules after a Blood Meal in the Asian Tiger Mosquito Aedes albopictus
Source: PLoS Negl Trop Dis. 2014 Jun 5;8(6):e2929. doi: 10.1371/journal.pntd.0002929 (PMC4046972; doi:10.1371/journal.pntd.0002929)

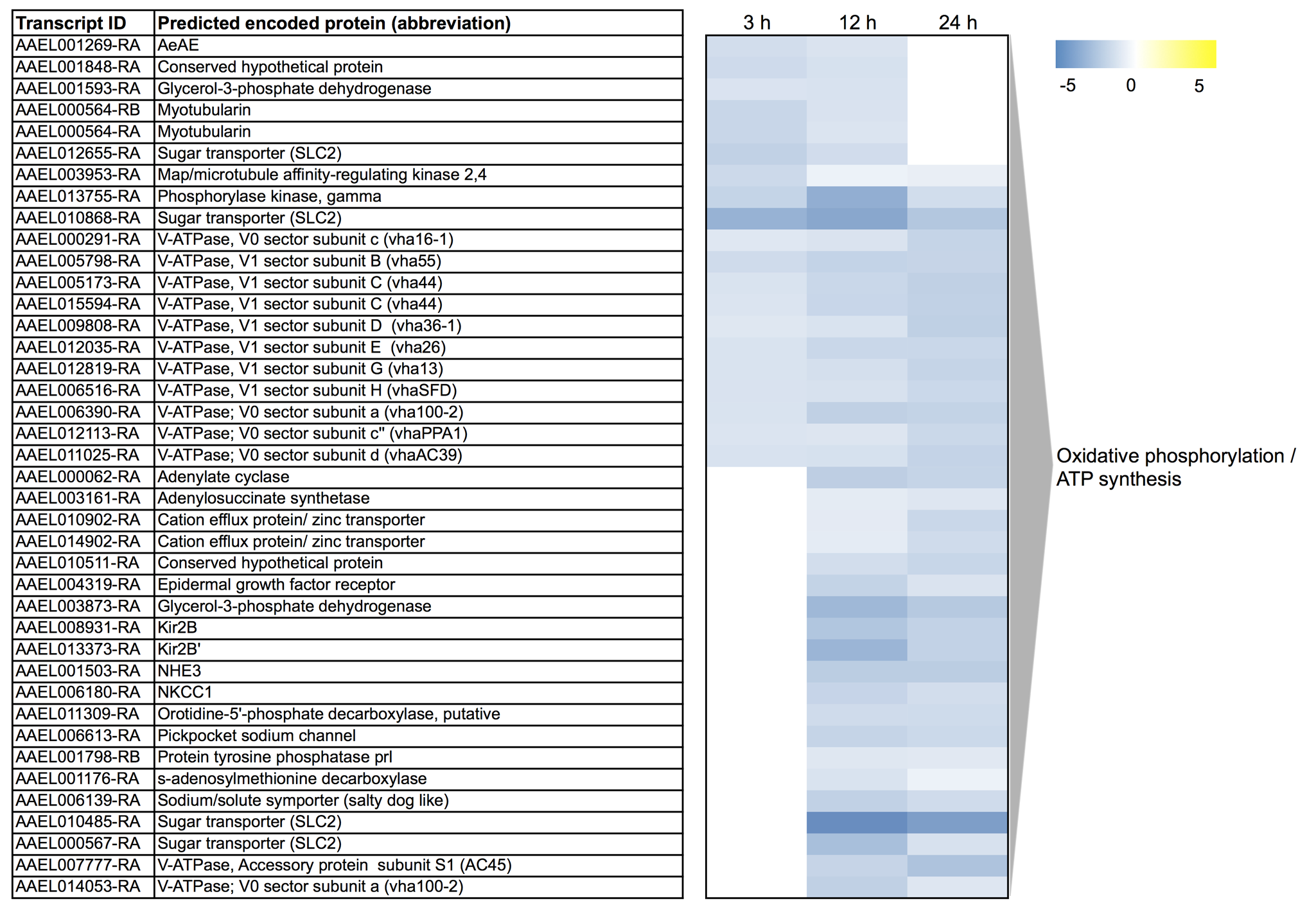

Supplement: Figure S1 — Lists of transcripts that exhibited a sustained down-regulation after blood feeding and were associated with the ‘oxidative phosphorylation/ATP synthesis’ DAVID functional cluster (see Table 2). Blue shading indicates significant down-regulation compared to NBF controls. Lack of shading indicates no significant difference relative to NBF controls. Degree of shading is based on value of Log2 fold change as indicated by the scale below on the right. (TIF) [file pntd.0002929.s001.tif]

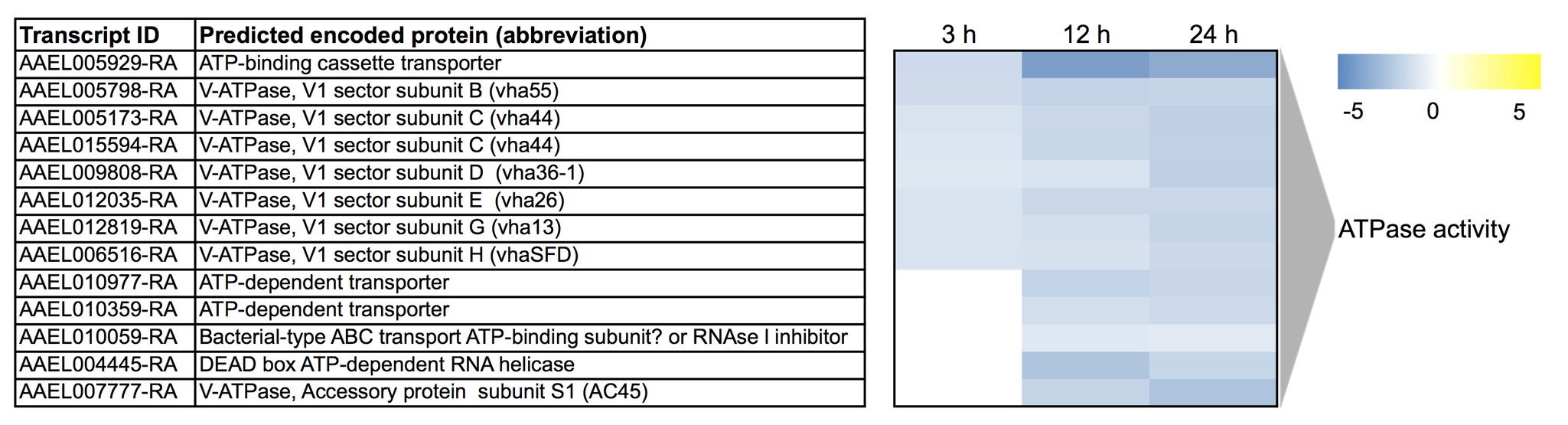

Supplement: Figure S2 — Lists of transcripts that exhibited a sustained down-regulation after blood feeding and were associated with the ‘ATPase activity’ DAVID functional cluster (see Table 2). Blue shading indicates significant down-regulation compared to NBF controls. Lack of shading indicates no significant difference relative to NBF controls. Degree of shading is based on value of Log2 fold change as indicated by the scale below on the right. (TIF) [file pntd.0002929.s002.tif]

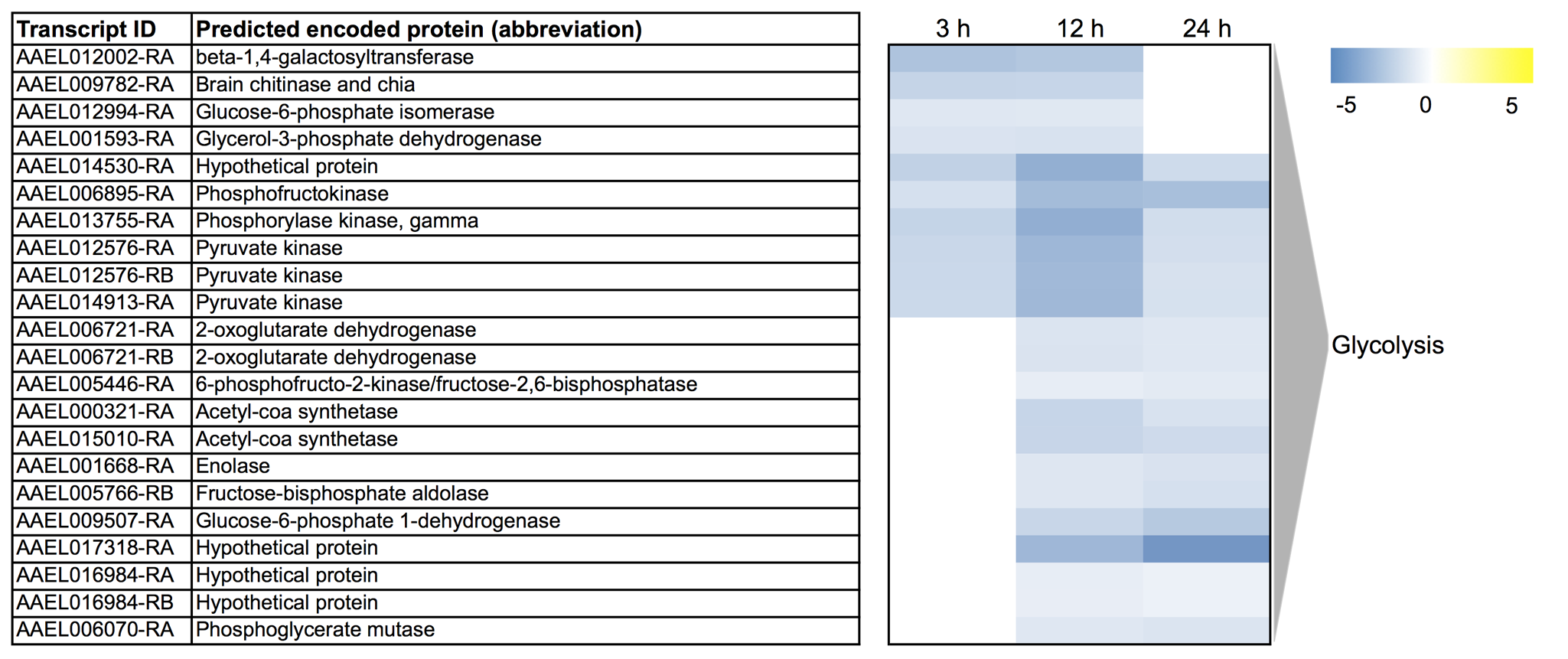

Supplement: Figure S3 — Lists of transcripts that exhibited a sustained down-regulation after blood feeding and were associated with the ‘glycolysis’ DAVID functional cluster (see Table 2). Blue shading indicates significant down-regulation compared to NBF controls. Lack of shading indicates no significant difference relative to NBF controls. Degree of shading is based on value of Log2 fold change as indicated by the scale below on the right. (TIF) [file pntd.0002929.s003.tif]

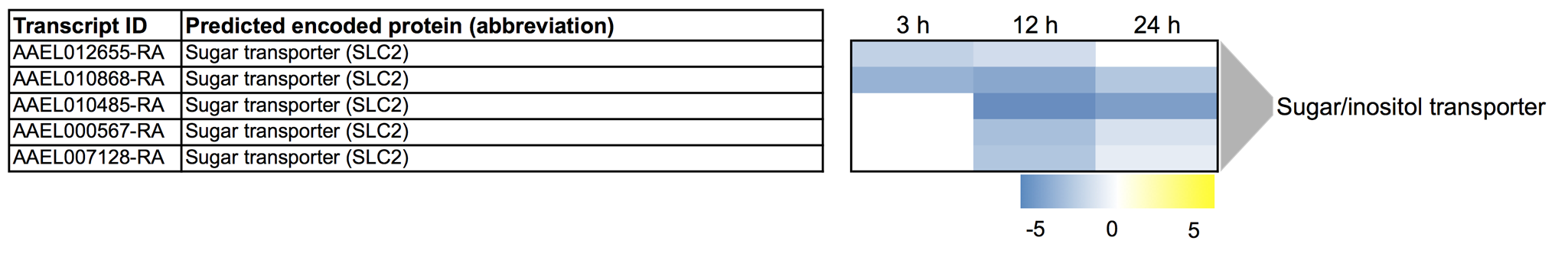

Supplement: Figure S4 — Lists of transcripts that exhibited a sustained down-regulation after blood feeding and were associated with the ‘sugar/inositol transporter’ DAVID functional cluster (see Table 2). Blue shading indicates significant down-regulation compared to NBF controls. Lack of shading indicates no significant difference relative to NBF controls. Degree of shading is based on value of Log2 fold change as indicated by the scale below on the right. (TIF) [file pntd.0002929.s004.tif]

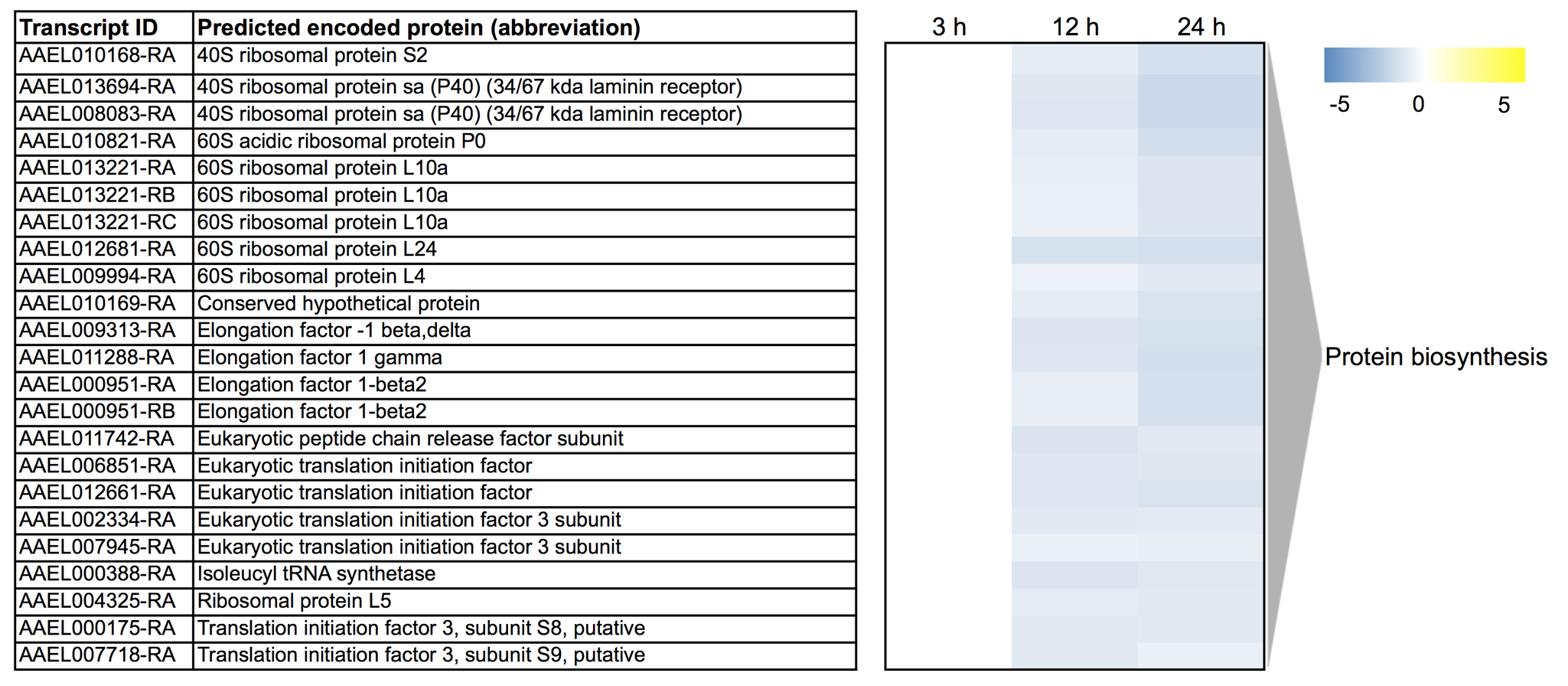

Supplement: Figure S5 — Lists of transcripts that exhibited a sustained down-regulation after blood feeding and were associated with the ‘protein biosynthesis’ DAVID functional cluster (see Table 2). Blue shading indicates significant down-regulation compared to NBF controls. Lack of shading indicates no significant difference relative to NBF controls. Degree of shading is based on value of Log2 fold change as indicated by the scale below on the right. (TIF) [file pntd.0002929.s005.tif]

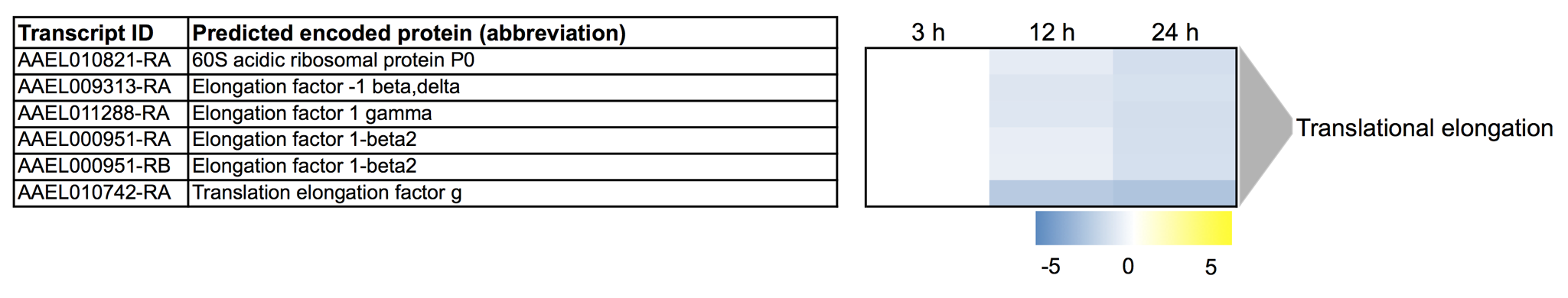

Supplement: Figure S6 — Lists of transcripts that exhibited a sustained down-regulation after blood feeding and were associated with the ‘translational elongation’ DAVID functional cluster (see Table 2). Blue shading indicates significant down-regulation compared to NBF controls. Lack of shading indicates no significant difference relative to NBF controls. Degree of shading is based on value of Log2 fold change as indicated by the scale below on the right. (TIF) [file pntd.0002929.s006.tif]

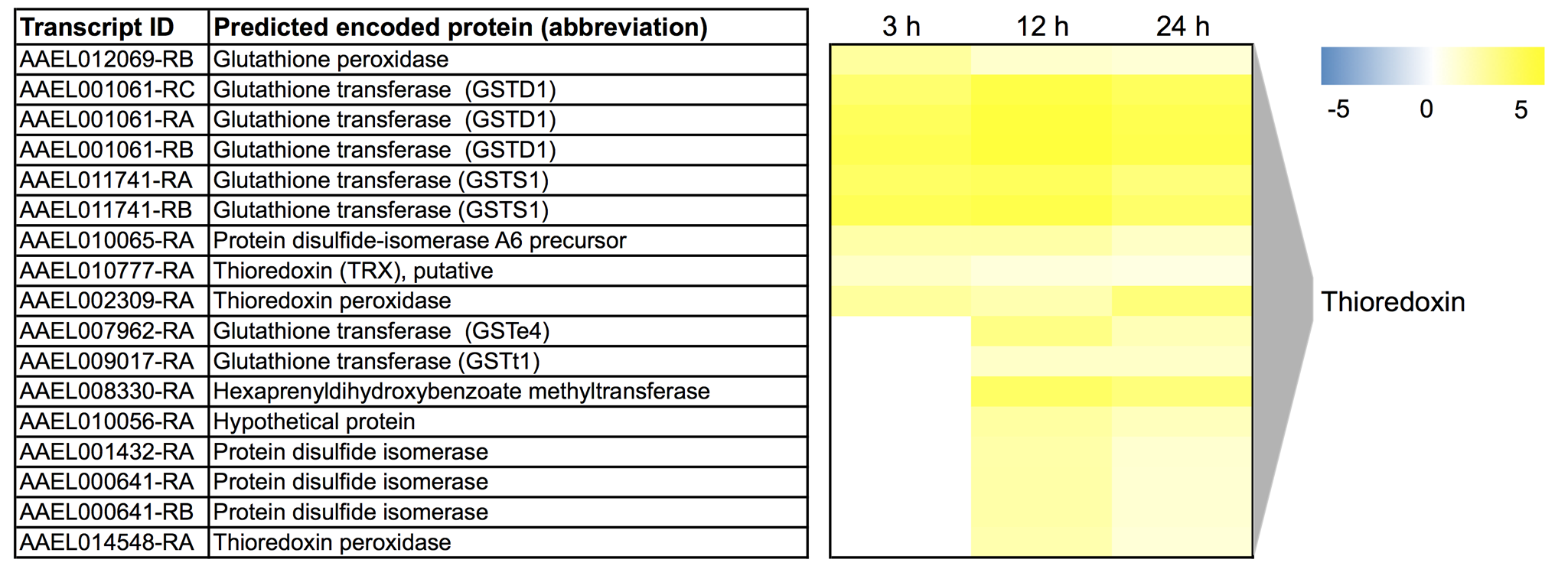

Supplement: Figure S7 — Lists of transcripts that exhibited a sustained up-regulation after blood feeding and were associated with the ‘thioredoxin’ DAVID functional cluster (see Table 2). Yellow shading indicates significant up-regulation compared to NBF controls. Lack of shading indicates no significant difference relative to NBF controls. Degree of shading is based on value of Log2 fold change as indicated by the scale below on the right. (TIF) [file pntd.0002929.s007.tif]

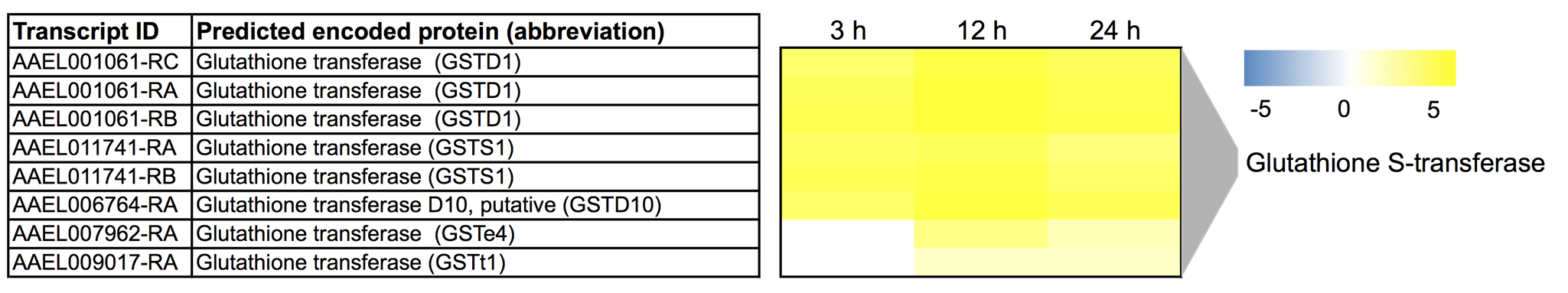

Supplement: Figure S8 — Lists of transcripts that exhibited a sustained up-regulation after blood feeding and were associated with the ‘glutathione S-transferase’ DAVID functional cluster (see Table 2). Yellow shading indicates significant up-regulation compared to NBF controls. Lack of shading indicates no significant difference relative to NBF controls. Degree of shading is based on value of Log2 fold change as indicated by the scale below on the right. (TIF) [file pntd.0002929.s008.tif]

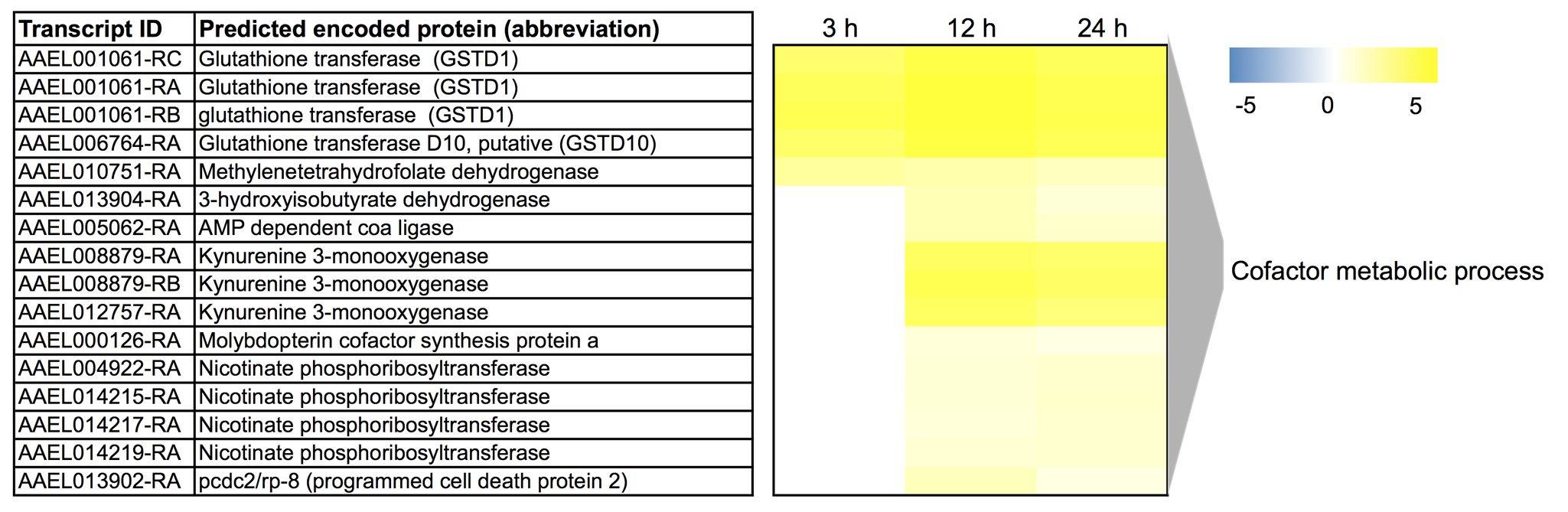

Supplement: Figure S9 — Lists of transcripts that exhibited a sustained up-regulation after blood feeding and were associated with the ‘cofactor metabolic process’ DAVID functional cluster (see Table 2). Yellow shading indicates significant up-regulation compared to NBF controls. Lack of shading indicates no significant difference relative to NBF controls. Degree of shading is based on value of Log2 fold change as indicated by the scale below on the right. (TIF) [file pntd.0002929.s009.tif]

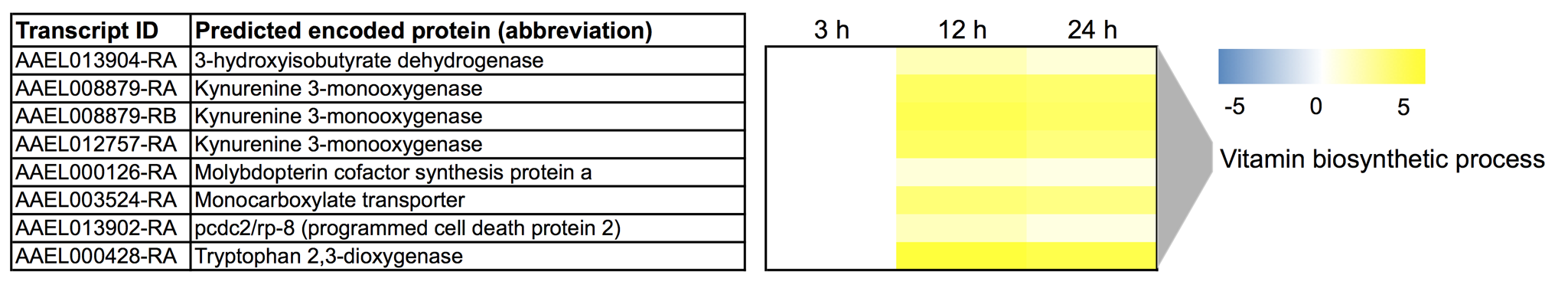

Supplement: Figure S10 — Lists of transcripts that exhibited a sustained up-regulation after blood feeding and were associated with the ‘vitamin biosynthetic process’ DAVID functional cluster (see Table 2). Yellow shading indicates significant up-regulation compared to NBF controls. Lack of shading indicates no significant difference relative to NBF controls. Degree of shading is based on value of Log2 fold change as indicated by the scale below on the right. (TIF) [file pntd.0002929.s010.tif]

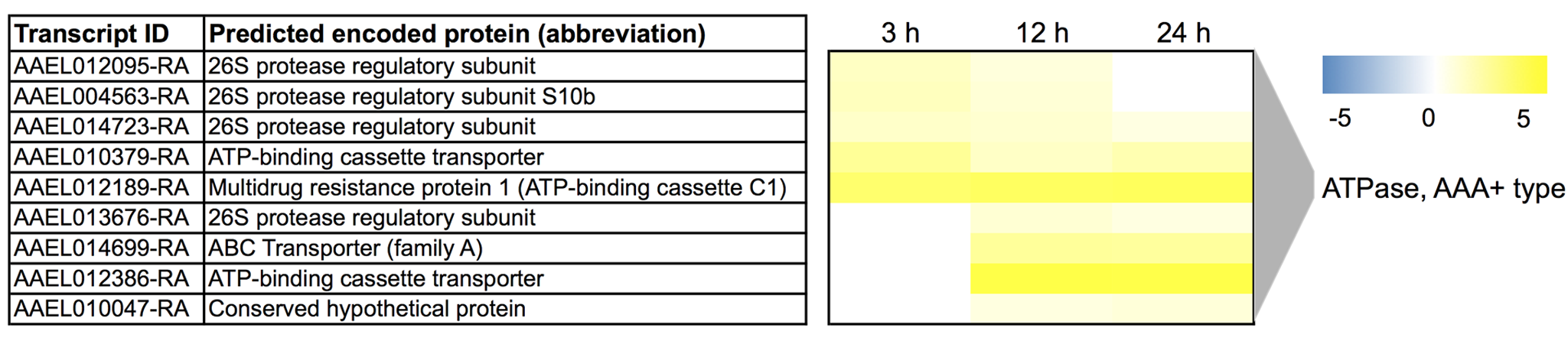

Supplement: Figure S11 — Lists of transcripts that exhibited a sustained up-regulation after blood feeding and were associated with the ‘ATPase, AAA+ type’ DAVID functional cluster (see Table 2). Yellow shading indicates significant up-regulation compared to NBF controls. Lack of shading indicates no significant difference relative to NBF controls. Degree of shading is based on value of Log2 fold change as indicated by the scale below on the right. (TIF) [file pntd.0002929.s011.tif]

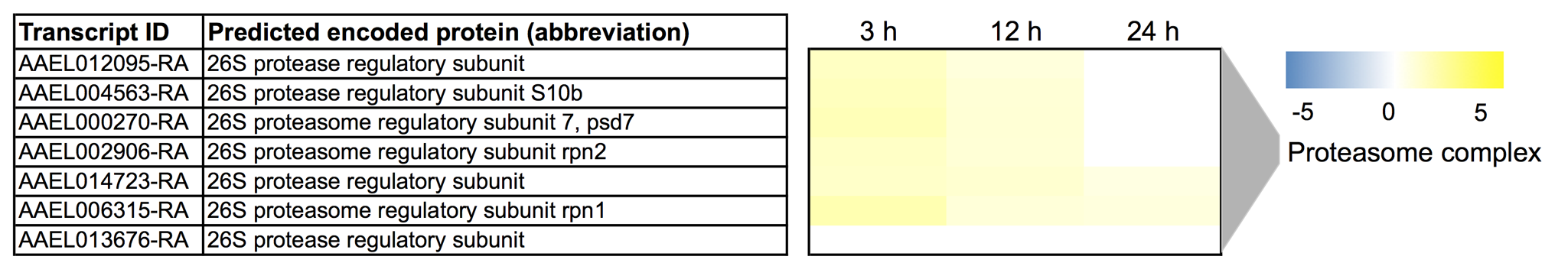

Supplement: Figure S12 — Lists of transcripts that exhibited a sustained up-regulation after blood feeding and were associated with the ‘proteasome complex’ DAVID functional cluster (see Table 2). Yellow shading indicates significant up-regulation compared to NBF controls. Lack of shading indicates no significant difference relative to NBF controls. Degree of shading is based on value of Log2 fold change as indicated by the scale below on the right. (TIF) [file pntd.0002929.s012.tif]

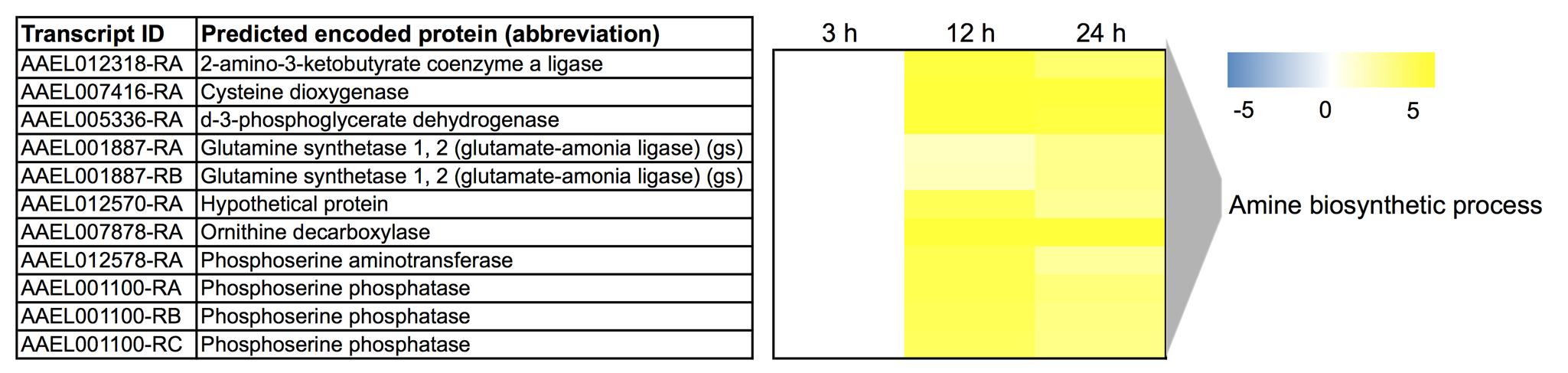

Supplement: Figure S13 — Lists of transcripts that exhibited a sustained up-regulation after blood feeding and were associated with the ‘amine biosynthetic process’ DAVID functional cluster (see Table 2). Yellow shading indicates significant up-regulation compared to NBF controls. Lack of shading indicates no significant difference relative to NBF controls. Degree of shading is based on value of Log2 fold change as indicated by the scale below on the right. (TIF) [file pntd.0002929.s013.tif]

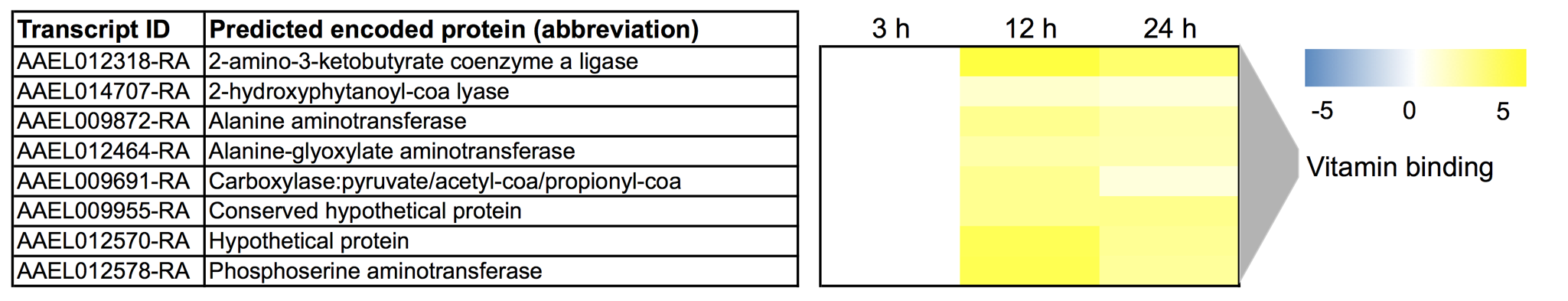

Supplement: Figure S14 — Lists of transcripts that exhibited a sustained up-regulation after blood feeding and were associated with the ‘vitamin binding’ DAVID functional cluster (see Table 2). Yellow shading indicates significant up-regulation compared to NBF controls. Lack of shading indicates no significant difference relative to NBF controls. Degree of shading is based on value of Log2 fold change as indicated by the scale below on the right. (TIF) [file pntd.0002929.s014.tif]

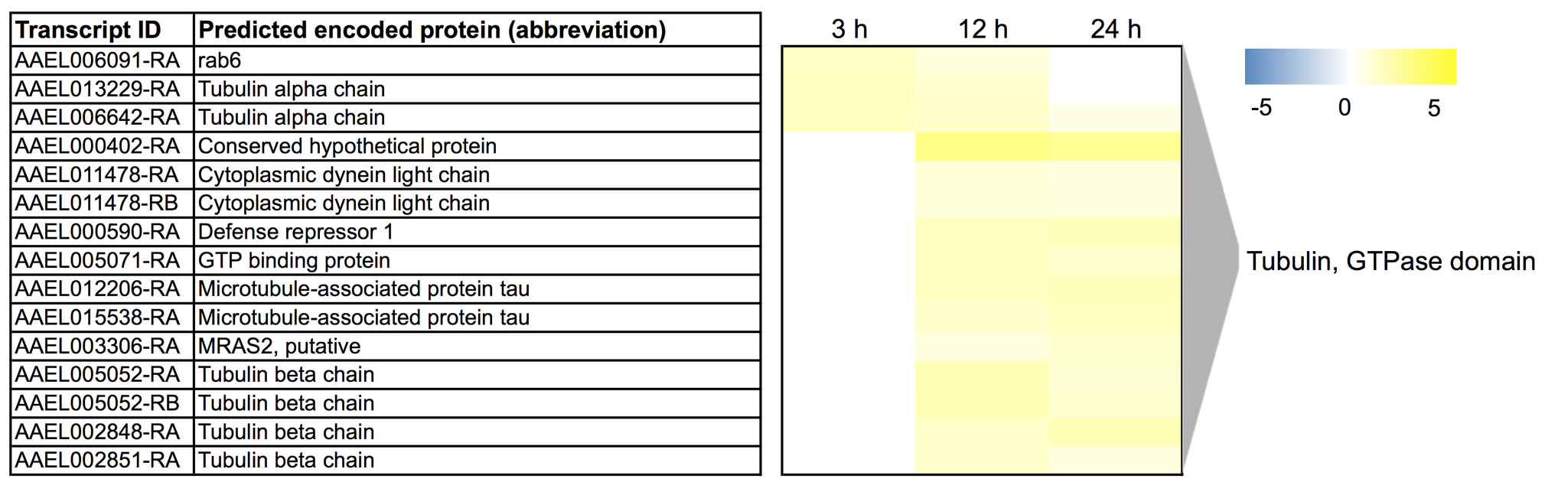

Supplement: Figure S15 — Lists of transcripts that exhibited a sustained up-regulation after blood feeding and were associated with the ‘tubulin, GTPase domain’ DAVID functional cluster (see Table 2). Yellow shading indicates significant up-regulation compared to NBF controls. Lack of shading indicates no significant difference relative to NBF controls. Degree of shading is based on value of Log2 fold change as indicated by the scale below on the right. (TIF) [file pntd.0002929.s015.tif]
