# Supplementary material for: Transcriptomic Evidence for a Dramatic Functional Transition of the Malpighian Tubules after a Blood Meal in the Asian Tiger Mosquito Aedes albopictus
Source: PLoS Negl Trop Dis. 2014 Jun 5;8(6):e2929. doi: 10.1371/journal.pntd.0002929 (PMC4046972; doi:10.1371/journal.pntd.0002929)
Supplement: Table S1 — Number of ‘raw reads’ and ‘reads mapped’ for the Malpighian tubule cDNA libraries that were sequenced using RNA-Seq. (DOCX) [file pntd.0002929.s016.docx]

| **Treatment** | **Replicate** | **Raw reads** | **Reads mapped on *Ae. aegypti*** | **Reads mapped on *Ae. albopictus*** |
| --- | --- | --- | --- | --- |
| 3 h BF | R1 | 14,442,939 | 2,557,844 | 10,768,655 |
|  | R2 | 10,646,668 | 2,375,272 | 7,613,432 |
|  | R3 | 12,704,688 | 2,262,705 | 9,532,327 |
| 3 h NBF | R1 | 8,958,354 | 1,645,650 | 6,696,370 |
|  | R2 | 15,504,459 | 3,189,267 | 11,574,079 |
|  | R3 | 13,742,632 | 2,813,117 | 10,436,155 |
| 12 h- BF | R1 | 9,956,966 | 1,563,244 | 7,498,591 |
|  | R2 | 12,438,610 | 1,944,155 | 9,274,228 |
|  | R3 | 12,652,639 | 2,048,462 | 9,531,233 |
| 12 h NBF | R1 | 13,783,840 | 2,784,336 | 10,446,772 |
|  | R2 | 12,848,082 | 2,673,686 | 9,860,903 |
|  | R3 | 11,535,312 | 2,168,639 | 8,598,422 |
| 24h BF | R1 | 15,069,241 | 2,194,081 | 11,119,593 |
|  | R2 | 12,818,006 | 1,913,728 | 9,418,671 |
|  | R3 | 15,098,776 | 2,304,073 | 11,203,292 |
| 24 h NBF | R1 | 9,268,704 | 1,840,765 | 6,878,305 |
|  | R2 | 18,016,970 | 3,680,867 | 13,763,163 |
|  | R3 | 12,957,001 | 2,714,492 | 10,000,213 |
| Total |  | 232,443,887 | 42,674,382 | 174,214,404 |
| Average |  | 12,913,549 | 2,370,799 | 9,678,578 |
