# Supplementary material for: Transcriptomic Evidence for a Dramatic Functional Transition of the Malpighian Tubules after a Blood Meal in the Asian Tiger Mosquito Aedes albopictus
Source: PLoS Negl Trop Dis. 2014 Jun 5;8(6):e2929. doi: 10.1371/journal.pntd.0002929 (PMC4046972; doi:10.1371/journal.pntd.0002929)
Supplement: Table S2 — ANOVA of non-blood fed libraries across time points. Number of reads per transcript per library was used as the independent variable. (DOCX) [file pntd.0002929.s017.docx]

| The SAS system, GLM procedure. | | | | | |
| --- | --- | --- | --- | --- | --- |
| *Source* | *DF^a^* | *Sum of squares* | *Mean square* | *F values* | *Pr > F* |
| Model | 533 | 6682246977 | 12537048.74 | 2.79 | <.0001 |
| Error | 168387 | 7.56617E+11 | 4493322.37 |  |  |
| Corrected total | 168920 | 7.63299E+11 |  |  |  |
|  |  |  |  |  |  |
| *Source* | *DF* | *Type I SS* | *Mean square* | *F values* | *Pr > F* |
| time | 2 | 2813970 | 1406985 | 0.31 | 0.7312 |
| transcripts | 177 | 6660816716 | 37631733 | 8.38 | <.0001 |
| time*transcripts | 354 | 18616291 | 52588 | 0.01 | 1 |
| ^a^Degrees of freedom | |  |  |  |  |
