# Supplementary material for: Transcriptomic Evidence for a Dramatic Functional Transition of the Malpighian Tubules after a Blood Meal in the Asian Tiger Mosquito Aedes albopictus
Source: PLoS Negl Trop Dis. 2014 Jun 5;8(6):e2929. doi: 10.1371/journal.pntd.0002929 (PMC4046972; doi:10.1371/journal.pntd.0002929)
Supplement: Table S3 — ANOVA of blood fed libraries across time points. Number of reads per transcript per library was used as the independent variable. (DOCX) [file pntd.0002929.s018.docx]

The SAS system, GLM procedure.

| *Source* | *DF^a^* | *Sum of Squares* | *Mean Square* | *F Value* | *Pr > F* |
| --- | --- | --- | --- | --- | --- |
| Model | 533 | 3162891904 | 5934131.151 | 2.88 | <.0001 |
| Error | 168387 | 3.47283E+11 | 2062411.956 |  |  |
| Corrected Total | 168920 | 3.50446E+11 |  |  |  |
|  |  |  |  |  |  |
| *Source* | *DF* | *Type I SS* | *Mean Square* | *F Value* | *Pr > F* |
| time | 2 | 16743581 | 8371790 | 4.06 | 0.0173 |
| transcripts | 177 | 3027679958 | 17105536 | 8.29 | <.0001 |
| time*transcripts | 354 | 118468365 | 334656 | 0.16 | 1 |
| ^a^Degrees of freedom | |  |  |  |  |
